# Salicylic acid modulates levels of phosphoinositide dependent-phospholipase C substrates and products to remodel the Arabidopsis suspension cell transcriptome

**DOI:** 10.3389/fpls.2014.00608

**Published:** 2014-11-11

**Authors:** Eric Ruelland, Igor Pokotylo, Nabila Djafi, Catherine Cantrel, Anne Repellin, Alain Zachowski

**Affiliations:** ^1^Université Paris-Est Créteil, Institut d'Ecologie et des Sciences de l'Environnement de ParisCréteil, France; ^2^Centre National de la Recherche Scientifique, Unité Mixte de Recherche 7618, Institut d'Ecologie et des Sciences de l'Environnement de ParisCréteil, France; ^3^Molecular Mechanisms of Plant Cell Regulation, Institute of Bioorganic Chemistry and Petrochemistry, National Academy of SciencesKyiv, Ukraine

**Keywords:** lipid signaling, salicylic acid, hormone transduction, Arabidopsis, phospholipase C, diacylglycerol kinase, trancriptomic

## Abstract

Basal phosphoinositide-dependent phospholipase C (PI-PLC) activity controls gene expression in Arabidopsis suspension cells and seedlings. PI-PLC catalyzes the production of phosphorylated inositol and diacylglycerol (DAG) from phosphoinositides. It is not known how PI-PLC regulates the transcriptome although the action of DAG-kinase (DGK) on DAG immediately downstream from PI-PLC is responsible for some of the regulation. We previously established a list of genes whose expression is affected in the presence of PI-PLC inhibitors. Here this list of genes was used as a signature in similarity searches of curated plant hormone response transcriptome data. The strongest correlations obtained with the inhibited PI-PLC signature were with salicylic acid (SA) treatments. We confirm here that in Arabidopsis suspension cells SA treatment leads to an increase in phosphoinositides, then demonstrate that SA leads to a significant 20% decrease in phosphatidic acid, indicative of a decrease in PI-PLC products. Previous sets of microarray data were re-assessed. The SA response of one set of genes was dependent on phosphoinositides. Alterations in the levels of a second set of genes, mostly SA-repressed genes, could be related to decreases in PI-PLC products that occur in response to SA action. Together, the two groups of genes comprise at least 40% of all SA-responsive genes. Overall these two groups of genes are distinct in the functional categories of the proteins they encode, their promoter *cis*-elements and their regulation by DGK or phospholipase D. SA-regulated genes dependent on phosphoinositides are typical SA response genes while those with an SA response that is possibly dependent on PI-PLC products are less SA-specific. We propose a model in which SA inhibits PI-PLC activity and alters levels of PI-PLC products and substrates, thereby regulating gene expression divergently.

## Introduction

Phospholipids are secondary messengers in plant signal transduction pathways that start with stimulus perception and lead to changes in gene expression that alter the growth, development and/or physiology of cells (Janda et al., 2013). *Arabidopsis thaliana* cell suspension cultures (ACSC) are an amenable, simplified model in which to study specific signaling mechanisms that would be too complex in plant tissues or organs. For instance, ACSC were recently used to study sugar signaling (Kunz et al., 2014), MAPK kinase and phosphatase signaling (Schweighofer et al., 2014), chitosan and galacturonide elicitor signaling (Ledoux et al., 2014), photooxidative damage (Gutiérrez et al., 2014), auxin transmembrane transport (Seifertová et al., 2014) and ion channel activity (Haapalainen et al., 2012). ACSC are easily labeled which is convenient for studying metabolic fluxes (Tjellström et al., 2012) like those in lipid phospholipid signaling. ACSC were used in this way to show that a drop in temperature activates both phospholipase C (PLC) and phospholipase D (PLD) pathways (Ruelland et al., 2002), while abscisic acid only activated PLD (Hallouin et al., 2002). We were also showed that stimulation of ASCS with the phytohormone salicylic acid (SA) leads to the rapid activation of PLD and to the production of phosphatidic acid (PA) (Krinke et al., 2009; Rainteau et al., 2012). When PLD-catalyzed PA production is inhibited in the presence of primary alcohols SA-induced gene expression is strongly disrupted, showing that PLD activity is needed to control SA-triggered transcriptome changes (Krinke et al., 2009). *PATHOGENESIS RELATED-1* (*PR-1*), a SA response marker gene related to SA's role in establishing systemic acquired resistance against a broad spectrum of pathogens (Malamy et al., 1990; Metraux et al., 1990; Durrant and Dong, 2004), is one of these PLD-dependent genes. Treating plants with SA also increases either the PA level or the PLD activity in *A. thaliana, Brassica napus*, and *Glycine max* (Profotova et al., 2006; Kalachova et al., 2012), so the signaling information gained from ASCS, although an artificial system, is directly relevant to whole plant physiology.

Phosphoinositide-dependent phospholipase C (PI-PLC) catalyses the hydrolysis of phosphoinositides into soluble phosphorylated inositol and into diacylglycerol (DAG) (Pokotylo et al., 2014). In Arabidopsis, PI-PLC enzymes are encoded by 9 genes. Some isoforms are more markedly expressed in response to drought, cold or salt stress (Pokotylo et al., 2014). PI-PLC are involved in plant adaptation to drought, heat, and cold conditions, as shown by pharmacological or reverse genetic approaches (Pokotylo et al., 2014; Ruelland et al., in press). Recently, we showed that a basal level of PI-PLC activity controlled the expression of a number of genes in ACSC (Djafi et al., 2013). In the same ACSC model, two phosphoinositide substrates of PI-PLC, phosphatidylinositol-4-phosphate (PI4P) and phosphatidylinositol-4,5-bisphosphate (PI-4,5-P_2_), are formed when a type-III phosphatidylinositol-4-kinase (PI4K) is activated by SA (Krinke et al., 2007). Mechanistically, it is not known how PI-PLC has a downstream effect on gene expression in ACSC.

Here our aim was to clarify the position of PI-PLC in the phospholipidic and genetic control of the ACSC transcriptome in the context of plant hormone responses, and more specifically the SA response. In our previous pharmacological approach, we applied edelfosine and U73122 to inhibit PI-PLC in ACSC and used microarrays to establish which genes were up or down regulated. Here we used this list of PI-PLC controlled genes as a signature in similarity searches against archived microarray data. No additional microarray data was generated here, but data mining and bioinformatics analysis of the inhibited PI-PLC signature allowed us to reassess and extend previous correlations between transcriptome changes and levels of PI-PLC substrates or products triggered by SA. The detection of two SA-regulated pools of genes, distinct in their promoters, regulations and functions, allows us to propose a new working model of the role of PI-PLC and phospholipid signaling in SA transduction, in which both PI-PLC products and substrates participate in SA-triggered transcriptome remodeling.

## Results

### Transcriptome changes controlled by edelfosine, a PI-PLC inhibitor, resemble those controlled by SA

We had previously identified genes whose basal expression is dependent on PI-PLC activity. Briefly, by microarray analysis, we monitored transcript levels in ACSC treated or not treated for 4 h with a PI-PLC inhibitor called edelfosine (Djafi et al., 2013). We defined the list of genes altered in response to edelfosine as a “signature.” The Genevestigator interface compares the transcript expression levels from an experiment of interest, the signature, to a subset of other microarray data and returns microarray experiments with similar gene responses. For each comparison, a correlation factor is calculated.

**Figure 1:**
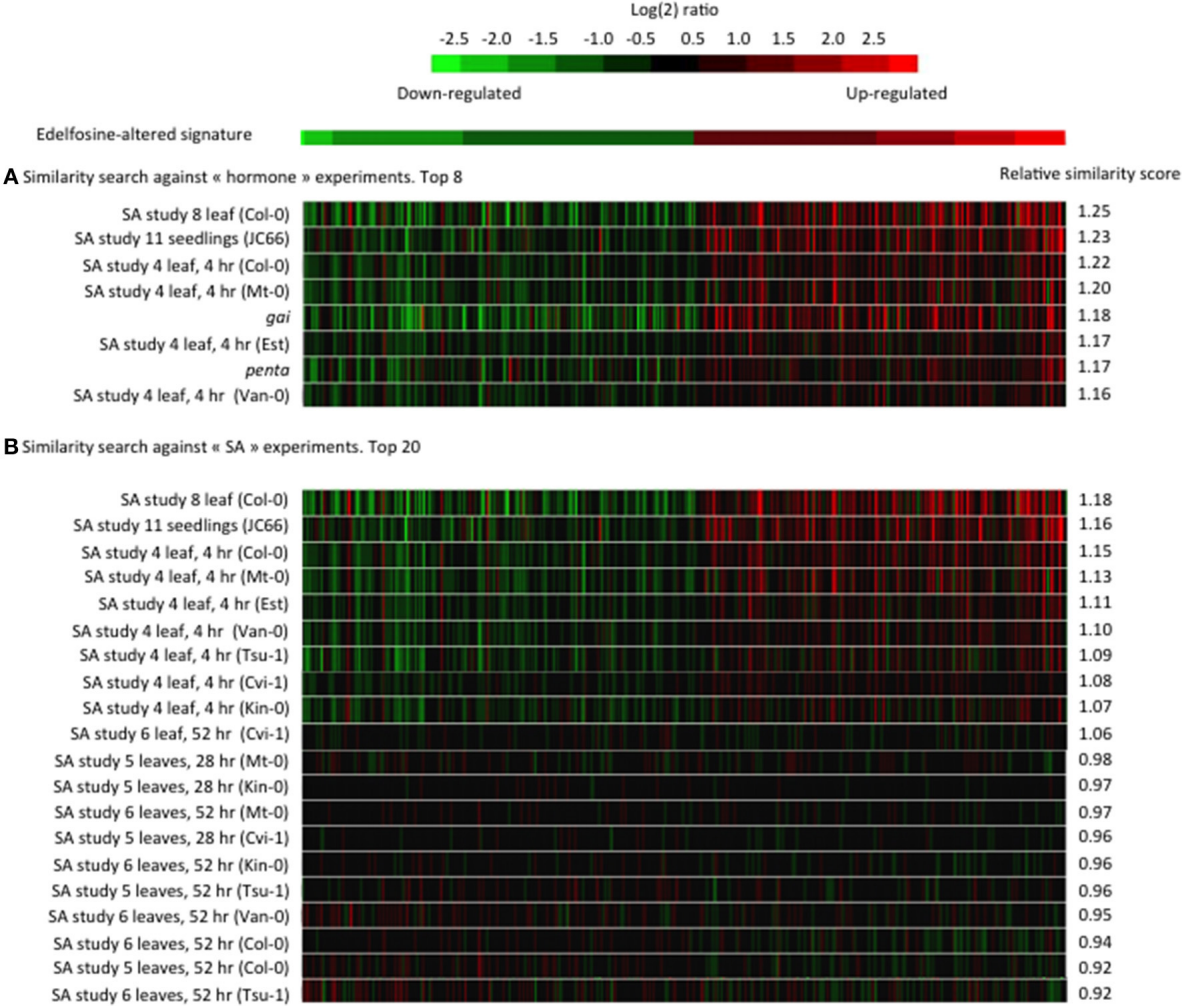
**Similarity between the edelfosine-responsive transcriptome and public transcriptome data**. The 200 genes the most up-regulated by edelfosine and the 200 genes the most down-regulated by edelfosine were used as a signature to search for experiments with similar transcriptome changes. A similarity score, derived from Euclidean distance, was calculated by Genevestigator (Hruz et al., 2008) between the edelfosine signature and each experiment of a set. Then a relative similarity score was calculated where a relative similarity score of 1 stands for a similarity between the input signature and an experiment that is the same as the average over all experiments of a set. **(A)** The similarity search was performed against the 734 experiments under the “hormone” classification. **(B)** The similarity search was performed against 155 SA-response experiments (GEO ID: AT_00494, AT_00557, and AT_00339). The relative similarity scores between our signature input and a particular experiment will be different in **(A,B)** because the overall sets of experiments are different.

From a subset of 734 microarray experiments categorized as dealing with phytohormone responses, the edelfosine-induced transcriptome changes in ACSC correlated most with changes caused by SA or by the *gai* and *penta* mutations (Figure 1A). SA is a major phytohormone well known for its action in plant responses to biotic stresses (Janda and Ruelland, in press). SA has other roles in plant responses to many abiotic stresses such as chilling, heat, heavy metal toxicity, drought, osmotic stress, and salinity (Horvath et al., 2007; Vicente and Plasencia, 2011). The *penta* mutant is mutated in genes encoding DELLA proteins, while *gai* is a constitutively active dominant DELLA mutant. DELLA proteins are central regulators of growth responses in gibberellin and light signaling pathways (Achard and Genschik, 2008). Interestingly, it was shown that DELLAs promote susceptibility to virulent biotrophs and resistance to necrotrophs, partly by altering the relative strength of SA and jasmonic acid signaling (Navarro et al., 2008). Even though we cannot rule out a direct crosstalk between edelfosine and DELLAs, most, if not all, of the top ranked transcriptome experiments have some link to SA signaling.

As the strongest and most frequent correlations were with the SA response, we performed a second signature similarity search, this time restricting the query database to experiments specifically dealing with SA responses (Figure 1B). The similarity between the edelfosine response and the SA gene response is found in several independent SA experiments on whole seedlings or leaves and in many Arabidopsis cultivars. We noted that the edelfosine data correlates more with the early SA response (4 h) than with later responses (28 or 52 h).

### Identification of genes similarly controlled by two PI-PLC inhibitors and SA in ACSC

In the past, we had analyzed the early SA-triggered transcriptome response in the same ACSC model as the one used for our more recent edelfosine experiments. The list of genes whose expression is altered after 4 h of incubation with SA is given in Table S1A (Krinke et al., 2007). We built a contingency table summarizing the results of the edelfosine and SA experiments by categorizing the responses of each individual gene. A gene may be up-regulated, down-regulated, or not affected by SA. Equally, a gene may be up-regulated, down-regulated, or not affected by edelfosine. An individual gene will therefore have one of nine possible response modes to SA and edelfosine (Table 1). From the microarray experiments, the response modes of 20556 genes were known and these observations were tabulated (Table 1).

**Table 1:** **Contingency table summarizing the gene expression response of Arabidopsis cells to edelfosine or SA treatments**.

**Number of transcripts per response mode**	**SA > control**	**SA = control**	**SA < control**	**Total**
Edelfosine > control	89	301	3	393
	(8.9)	(377.9)	(6.1)	
	**10**	**0.8**	**0.5**	
Edelfosine = control	365	19,197	175	19,737
	(450.1)	(18,980.8)	(306.1)	
	**0.8**	**1.0**	**0.6**	
Edelfosine < control	15	280	141	436
	(9.9)	(419.3)	(6.8)	
	**1.5**	**0.7**	**21**	
Total	469	19,778	319	20,556

*For each response mode, the number of genes previously reported to be up-regulated (>), down-regulated (<) or unaffected (=) by a treatment were included. Assuming that edelfosine and SA act independently on gene expression, the number of genes predicted to be found for each response mode (given in brackets) was calculated as the product of the corresponding row and column totals divided by the total number of genes tested. The ratio of the “observed number of genes” to “theoretical number of genes” is given in bold*.

To investigate the observed response modes of genes, we first supposed that the effects of SA and edelfosine were independent. Knowing the total number of genes tested on the microarrays, and the total number of genes up-regulated, down-regulated, or unchanged by each treatment separately, it is possible to calculate how many genes would theoretically belong to each of the 9 response modes if they were sorted randomly. We calculated the ratios between the observed number of genes per response mode and the theoretical one (Table 1). We identified 89 genes that are induced both by SA and edelfosine treatment, and 156 genes that are repressed by both treatments. This is not a random distribution as there are 10-fold more genes induced by SA and edelfosine (separately) than would be expected in a random distribution, while 20-fold more were repressed by both treatments than would be expected. On the contrary, the genes on which SA and edelfosine have opposite effects are not over-represented.

To demonstrate that the relationship between PI-PLC inhibition and SA action was not due to non-specific effects of edelfosine, we used another PI-PLC inhibitor, U73122, which is chemically different from edelfosine (Djafi et al., 2013). We made another contingency table cross-categorizing the list of SA-responsive genes with the list of U73122-responsive genes (Table S2). Again a statistical over-representation of genes similarly regulated by SA and U73122 was observed. Genes induced separately by both SA and U73122 are 7-fold more frequent than would be expected for a random distribution, while those repressed separately by both SA and U73122 are 12-fold more frequent. The genes that respond in the same way to SA and edelfosine are listed in Table S1B and those that respond in the same way to SA and U73122 are listed in Table S1C.

We built a third contingency table categorizing the separate effects of edelfosine and U73122 with their nine possible gene response modes, relative to the three possible SA response modes, giving 27 possible response modes in total (Table 2). The genes that are induced by U73122 and by edelfosine are statistically more likely to be SA-inducible than would be expected if the effects were independent. The genes that are induced by edelfosine but unaffected by U73122, and the genes that are induced by U73122 but unaffected by edelfosine are also more likely to be SA inducible. The over-representation effect is more marked for genes induced by both inhibitors (12.8-fold) than for genes induced by only one inhibitor (i.e., 4.1-fold for U73122-induced and 7.7-fold for edelfosine-induced genes). On the contrary, SA-inhibited genes are less common than expected among the genes that are induced by either one of the two PI-PLC inhibitors. The genes that are repressed by U73122 and by edelfosine are statistically more likely to be SA-repressed than would be expected if the effects were independent. The genes that are repressed only by edelfosine and the genes that are repressed only by U73122 are also more likely to be SA-repressed. The over-representation effect is more marked for genes repressed by both inhibitors (29.6-fold) than for genes repressed by only one of them (3.5-fold for U73122-induced genes and 9.3-fold for edelfosine-induced genes). The list of genes that respond in the same way to SA, edelfosine and U73122 is in Table S1D.

**Table 2:** **Contingency table summarizing the gene expression response of Arabidopsis cells to edelfosine, U73122 or SA treatments**.

**Number of transcripts per response mode**	**SA > control**	**SA = control**	**SA < control**	**Total**
Edelfosine > control	60	147	1	208
U73122 > control	(4.7)	(200.1)	(3.2)	
	**12.8**	**0.7**	**0.31**	
Edelfosine = control	119	1238	4	1361
U73122 > control	(28.9)	(1309.2)	(21.1)	
	**4.1**	**0.9**	**0.19**	
Edelfosine > control	28	131	2	161
U73122 = control	(3.6)	(154.9)	(2.5)	
	**7.7**	**0.8**	**0.8**	
Edelfosine > control	2	4	0	6
U73122 < control	(0.13)	(5.8)	(0.1)	
	**14.8**	**0.7**	**0**	
Edelfosine = control	227	16558	89	16874
U73122 = control	(380)	(16232.2)	(261.8)	
	**0.6**	**1.02**	**0.34**	
Edelfosine > control	0	2	0	2
U73122 < control	(0.04)	(1.9)	(0.03)	
	**0**	**1.03**	**0**	
Edelfosine = control	14	1401	82	1497
U73122 < control	(33.7)	(1440.1)	(23.2)	
	**0.4**	**1.0**	**3.5**	
Edelfosine < control	8	171	30	209
U73122 = control	(4.7)	(201.1)	(3.2)	
	**1.7**	**0.9**	**9.3**	
Edelfosine < control	5	126	111	242
U73122 < control	(5.45)	(232.8)	(3.8)	
	**0.91**	**0.54**	**29.6**	
Total	463	19778	319	20560

*For each response mode, the number of genes previously reported to be up-regulated (>), down-regulated (<) or unaffected (=) by a treatment were included. Assuming that edelfosine and U73122 act independently of SA, the number of genes predicted to be found for each response mode (given in brackets) was calculated as the product of the corresponding row and column totals divided by the total number of genes tested. The ratio of the “observed number of genes” to the “theoretical number of genes” is given in bold*.

### SA inhibits basal PI-PLC activity in ACSC

**Figure 2:**
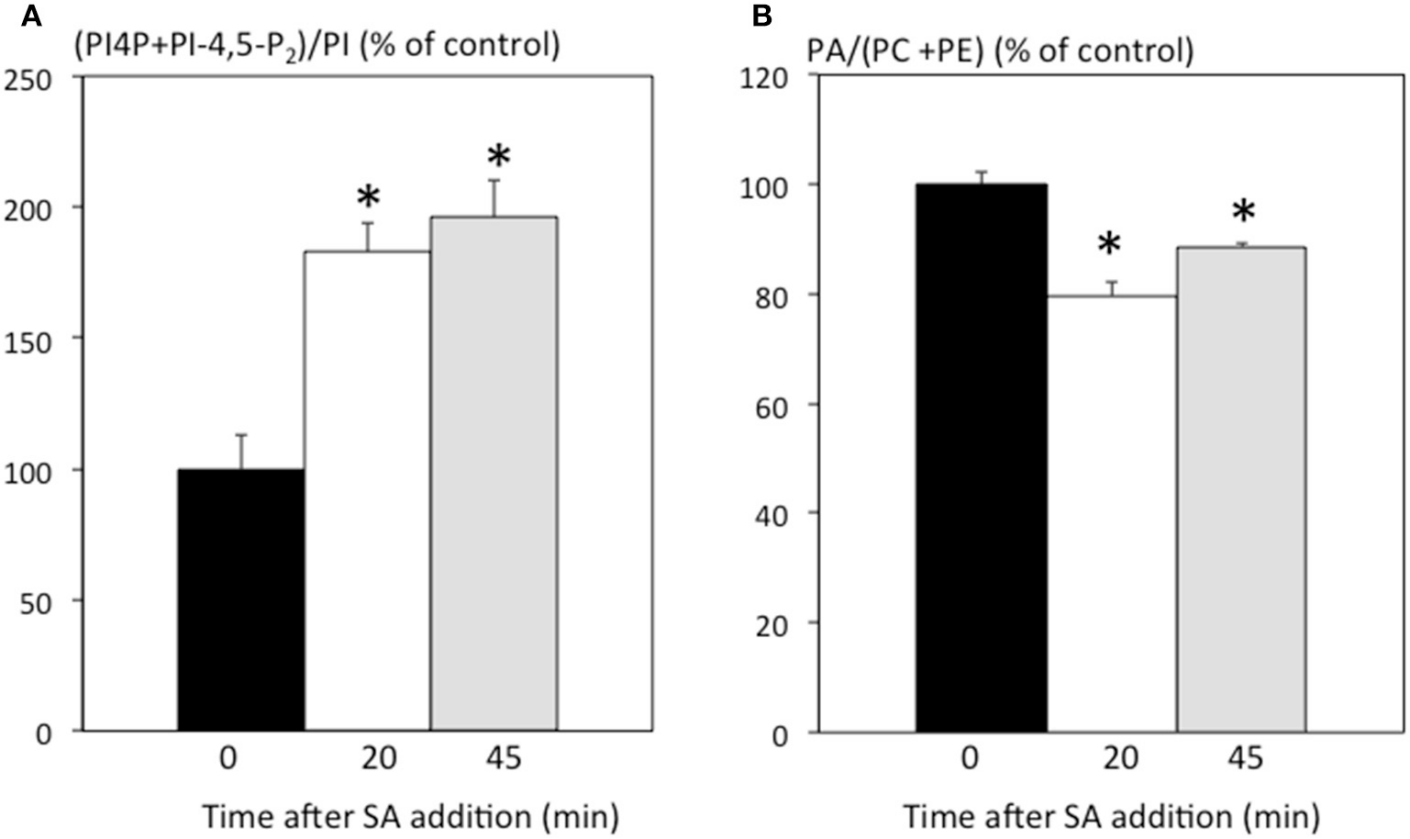
**SA effects on radioactively labeled phospholipids**. Cells were treated with 250 μM SA. Labeling was initiated 15 min before lipid extraction. Lipids were extracted and separated by TLC. **(A)** Amount of radioactivity incorporated into phosphoinositides relative to PI expressed as % of the control without SA. **(B)** Amount of radioactivity incorporated into PA relative to the sum of PC and PE expressed as % of the control without SA. ^*^Indicates a value statistically different from time 0 (*t*-test, *p*-value < 0.05).

The fact that SA treatment can be in part mimicked by inhibitors of PI-PLC might indicate that part of the SA signal is transduced via PI-PLC inhibition. To investigate whether PI-PLC is inhibited in ACSC, we used radioactive ^33^Pi orthophosphate labeling to visualize phosphorylated lipids. PI-PLCs use phosphoinositides PI4P and/or PI-4,5-P_2_ as substrates and release phosphorylated inositol and DAG, which can be further phosphorylated into PA by DGKs. Inhibition of PI-PLC would be expected to lead to an increase in substrates and a decrease in products (and of any derivatives). We added 250 μM SA to ACSC and extracted lipids 20 and 45 min later, labeling cells 15 min before the lipid extraction. When short labeling times are used, labeled PA is almost exclusively due to DGK activity (Vaultier et al., 2006; Djafi et al., 2013). As already reported (Krinke et al., 2007), SA induced an increase in phosphoinositides (Figure 2A). Furthermore, SA led to a small but significant decrease in PA (Figure 2B). This suggests that PI-PLC activity is inhibited as early as 20 min after SA stimulation, resulting in an increase in phosphoinositides and a decrease in DAG, PA and phosphorylated inositol.

### Are any SA-regulated genes regulated through PI-PLC inhibition and modulations in the levels of its substrates or products?

**Figure 3:**
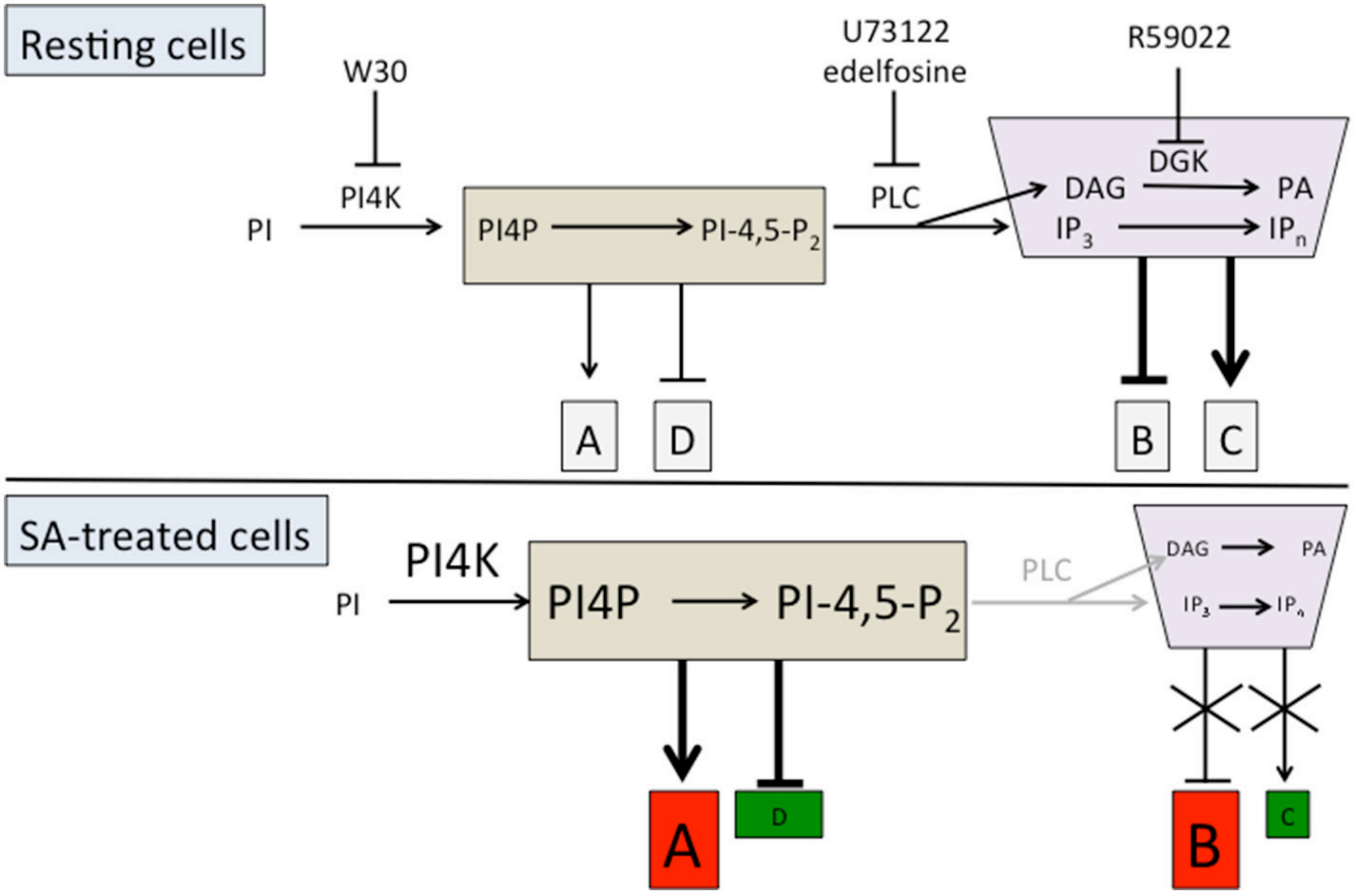
**Working model of the action of PI-PLC substrates and products on SA-triggered gene expression**. The action of PI-PLC substrates and products on gene expression is represented either by an arrow (positive action) or a line with a bar (negative action). The clusters of genes induced after SA treatment are represented in red and those inhibited in green.

We can hypothesize that PI-PLC substrates positively or negatively regulate the basal expression of clusters of genes in resting cells, named clusters A and D respectively in Figure 3. Similarly, PI-PLC products could negatively or positively regulate the expression of some genes, respectively clusters B and C (Figure 3). When SA inhibits PI-PLC activity, the resulting increase in substrates leads to an enhancement of their basal action on gene expression. Cluster A genes would be induced by SA and cluster D genes repressed by SA in a phosphoinositide-dependent manner. On the other hand, PI-PLC inhibition would alleviate the effects of its products on gene expression. Thus, cluster B genes would be induced and cluster C genes repressed compared to the resting state.

Is it possible to identify these hypothetical gene clusters? We have previously done a microarray experiment to find genes that are regulated by wortmannin. Type III-PI4Ks responsible for phosphoinositide formation are inhibited by 30 μM wortmannin (W30), but not 1 μM wortmannin (W1). These PI4Ks act immediately upstream of basal PI-PLC activity (Figure 3; Delage et al., 2012). For the genes that are regulated by basal PI-PLC products, W30 and edelfosine should have the same effects as both reduce the quantity of PI-PLC products. On the contrary, for the genes that are regulated by PI-PLC substrates, W30 and edelfosine should have opposite effects. W30 will diminish the level of phosphoinositides, while inhibiting PI-PLC with edelfosine will increase it.

To identify SA responsive genes regulated by PI-PLC inhibition, that is through substrate increase or product decrease, we can categorize the genes according to how they are regulated by SA, by edelfosine, by W30, or by SA in the presence of W30.

**Figure 4:**
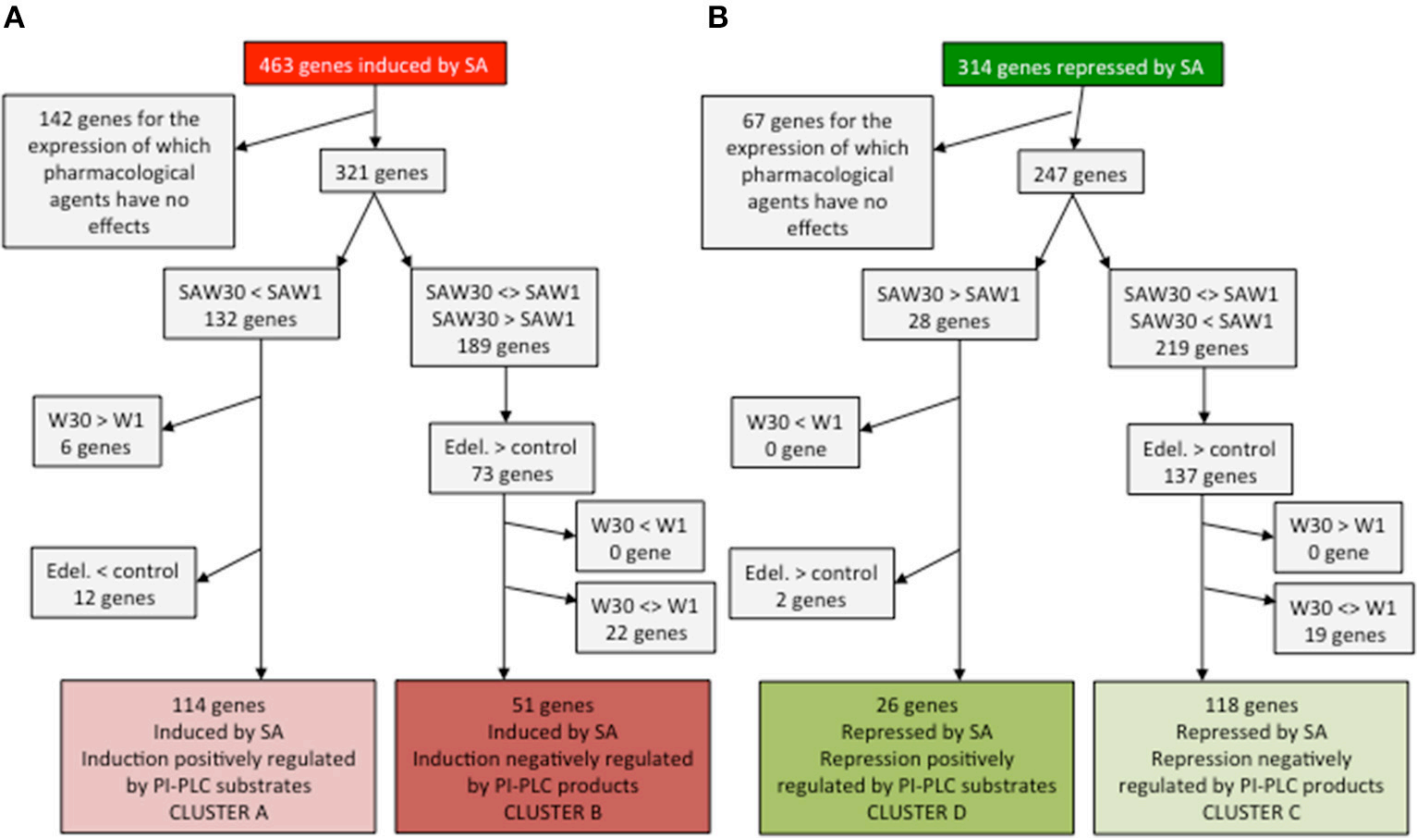
**Identification of genes whose expression characteristics are consistent with the clusters defined in Figure 3**. Cluster A and B genes are SA-induced genes **(A)** while clusters C and D genes are SA-repressed genes **(B)**.

From the 463 SA-induced genes in ACSC, we first discounted the 141 genes that were unaffected by treatments with edelfosine or W30 (Figure 4A). SA induction of cluster A genes is inhibited by W30 and not by W1, so they must be among the subset that is down-regulated in the SAW30 (SAW, SA + wortmannin) condition compared to the SAW1 condition. The basal level of regulation by PI-PLC substrates is already in place in resting cells, with SA-dependent PI-PLC inhibition only enhancing it. This basal regulation must be of the same nature and cannot be opposite to regulation in the presence of SA. The genes must be expressed at a lower level in W30 than in W1, so we can eliminate the 6 genes that are more expressed in W30 than in W1. The subset of 12 genes that are repressed by edelfosine is excluded, as this would not be compatible with basal PI-PLC substrates having a positive role. The remaining 114 genes thus form cluster A (Figure 4A). Cluster B genes are induced by an alleviation of repression via PI-PLC products. These genes must be induced by edelfosine and by W30, as both molecules inhibit the basal PI-PLC pathway. The effect of W30 in the presence of SA is either neutral or additive, so the 2 genes that are repressed by SAW30 compared to SAW1 can be excluded. Therefore, 51 genes have the characteristics of cluster B genes.

The same step-wise analysis can be made for SA-repressed genes (Figure 4B). Discounting 67 genes that show no alteration in expression with any treatment, 247 genes remain. Cluster D genes are repressed by SA through the action of PI-PLC substrates. They have higher expression in SAW30 than in SAW1. This regulation may be in place in the absence of SA, but cannot be oppositely regulated. We can thus identify 26 genes that have the characteristics of cluster D. A final important point is that the genes of cluster C are already regulated in resting cells as they are induced by a basal PI-PLC activity through its products. Edelfosine should repress their expression (149 remaining genes), and so should W30. The remaining 118 genes have the characteristics of cluster C genes. Genes in clusters A, B, C, and D are listed in Table S3.

**Figure 5:**
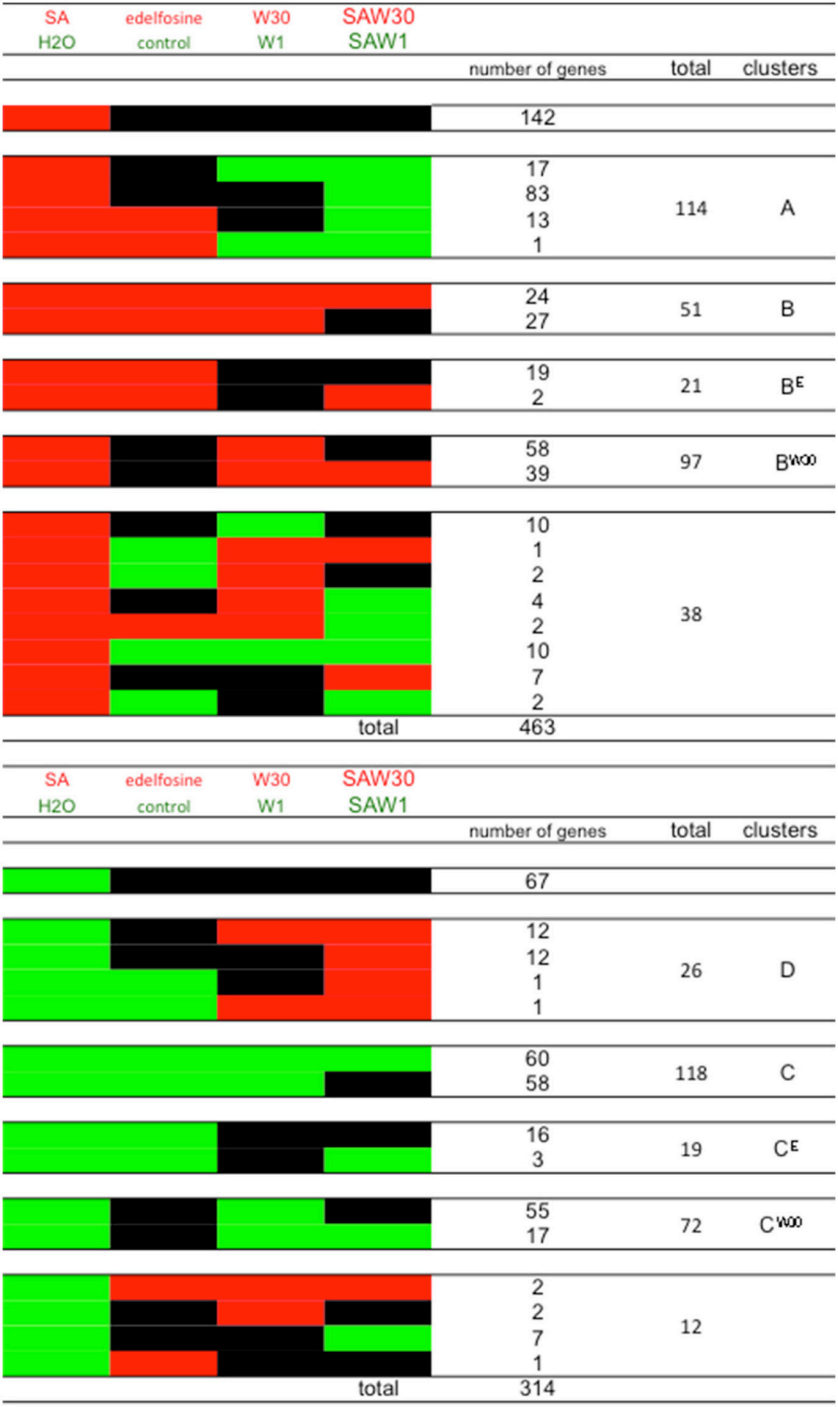
**Representation of genes of cluster A, B, C, and D according to their expression in response to SA, to edelfosine, to W30 and to W30 in the presence of SA (SAW30)**. Red blocks indicate relative higher transcript levels in the condition written in red at the top of the table (versus that written in green); green blocks indicate higher transcript levels in the conditions written green at the top of the table (versus that written in red) and black blocks indicate no significant difference in transcript levels between both conditions. Note that clusters B^E^ and C^E^ and clusters B^W30^ and C^W30^ are also represented here. Clusters B^E^ and B^W30^ are the genes that would belong to cluster B if we had considered that a basal inhibiting effect of either edelfosine or W30 respectively was sufficient for a gene to be classed as cluster B. Clusters C^E^ and C^W30^ are the genes that would belong to cluster B if we had considered that a basal inhibiting effect of either edelfosine or W30 respectively was sufficient for a gene to be classed as cluster C. These clusters are mentioned in Discussion.

The expression characteristics of the genes in these newly defined clusters can be visualized graphically in Figure 5. Cluster A genes are SA-induced genes that can or cannot be induced independently by edelfosine. Importantly, the induction by SA of these genes is repressed by W30. This inhibiting effect of W30 can also be detected on the basal expression without SA. Cluster B gene expression is induced by SA through the alleviation of a basal repression by PI-PLC products. These genes are induced independently by edelfosine and W30. For SA-repressed genes, the repression of cluster D genes is inhibited by W30, and inhibition by W30 can also, but not necessarily, be observed in resting cells. Cluster C genes are repressed by SA through alleviation of induction by PI-PLC products. W30 and edelfosine can repress cluster C expression in the absence of SA.

**Figure 6:**
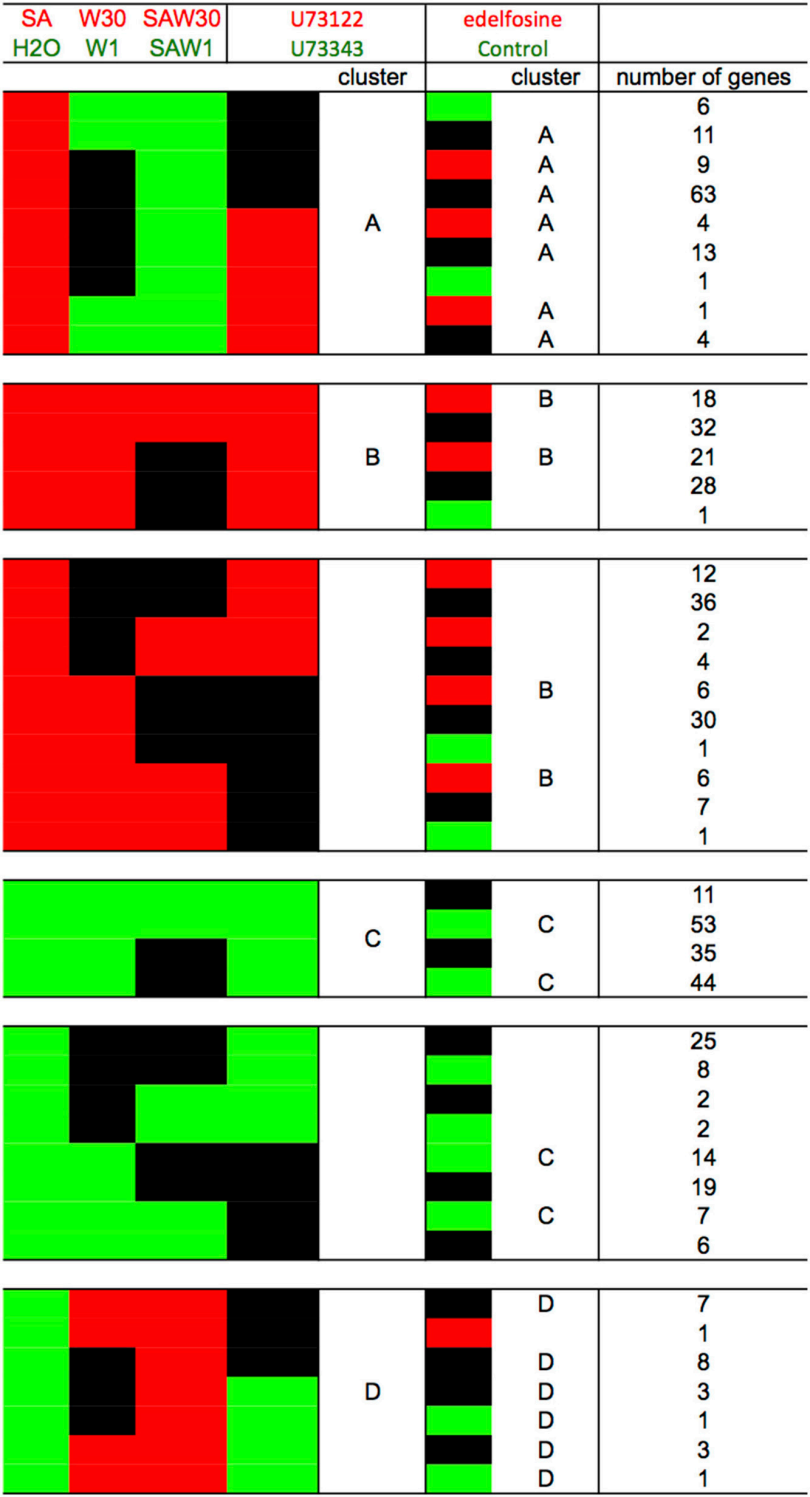
**Comparison of the clustering result according to the use of edelfosine or U73122 as the PI-PLC inhibitor**. Red blocks indicate relative higher transcript levels in the condition written in red at the top of the table (versus that written in green); green blocks indicate higher transcript levels in the conditions written green at the top of the table (versus that written in red) and black blocks indicate no significant difference in transcript levels between both conditions. Stringent clusters were defined from this analysis.

To pinpoint the effects of PI-PLC inhibition, we compared the effect of U73122 on gene expression to the effect of edelfosine to see if the same genes were assigned to each cluster despite using inhibitors with different biochemical modes of action (Figure 6). To continue the analysis, only the genes that were common to a cluster in both the edelfosine and the U73122 analyses were considered. In these *stringent* clusters A, B, C, and D, there are 105, 39, 97, and 23 genes, respectively (Table S4).

**Figure 7:**
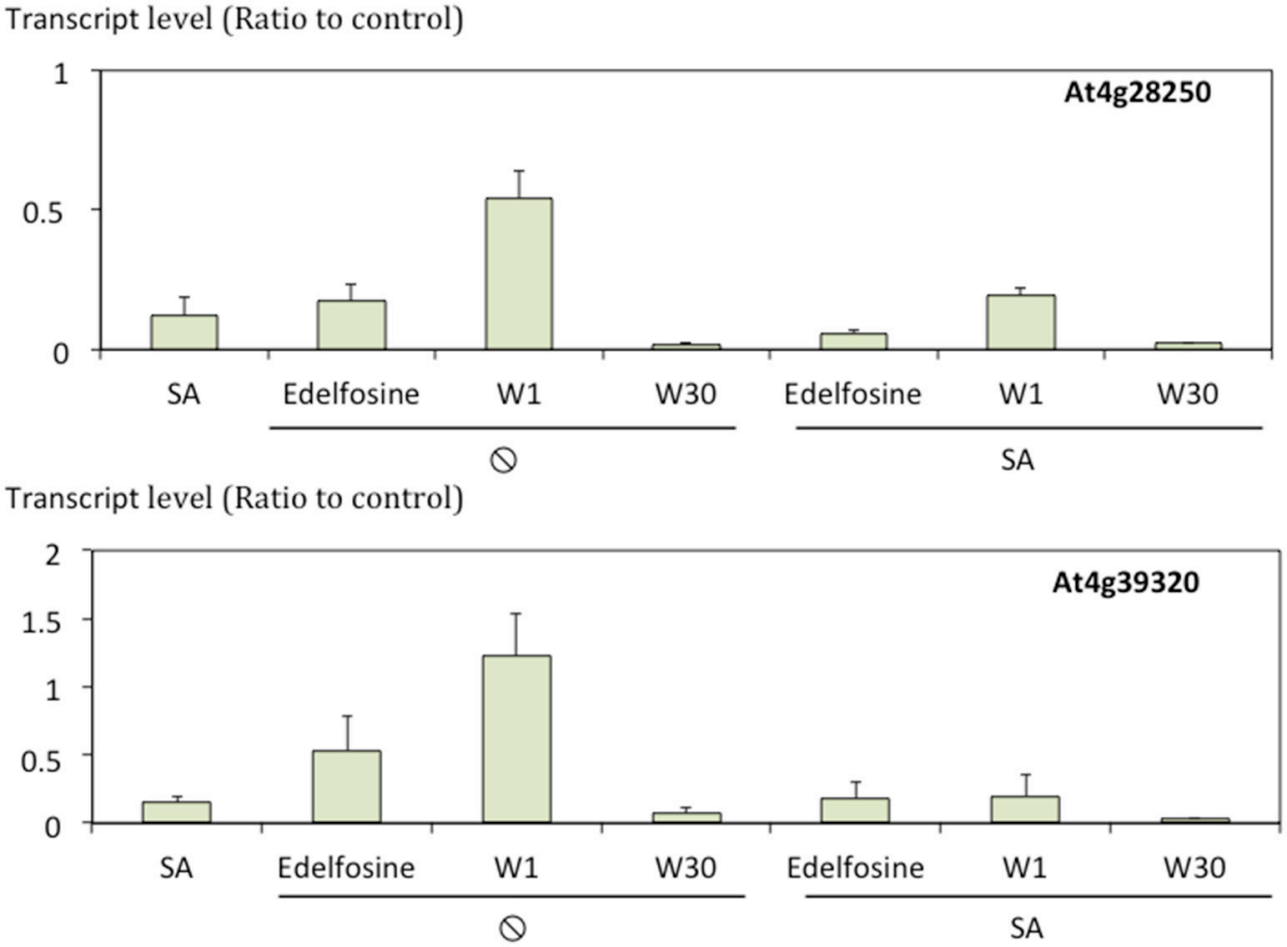
**Transcript levels of At4g28250 and At4g39320 in response to SA and/or lipid signaling inhibitors**. Transcript levels were quantified by qPCR, normalized to actine transcript level and expressed relative to levels in control cells. *n* = 3.

As all the data were from microarray experiments, the regulation of expression of some genes was confirmed by quantifying transcripts by qPCR. In fact, cluster A and cluster D gene expression had already been “confirmed” by Krinke et al. (2007). We therefore focused on two SA inhibited genes representative of the 118 genes in cluster C, the largest non-stringent cluster (Figure 7).

The SA responsive genes form two pools, one corresponding to genes dependent on PI-PLC substrates for their SA response, and the other corresponding to genes dependent on PI-PLC products for their SA response.

### Clusters include genes from specific functional classes

**Figure 8:**
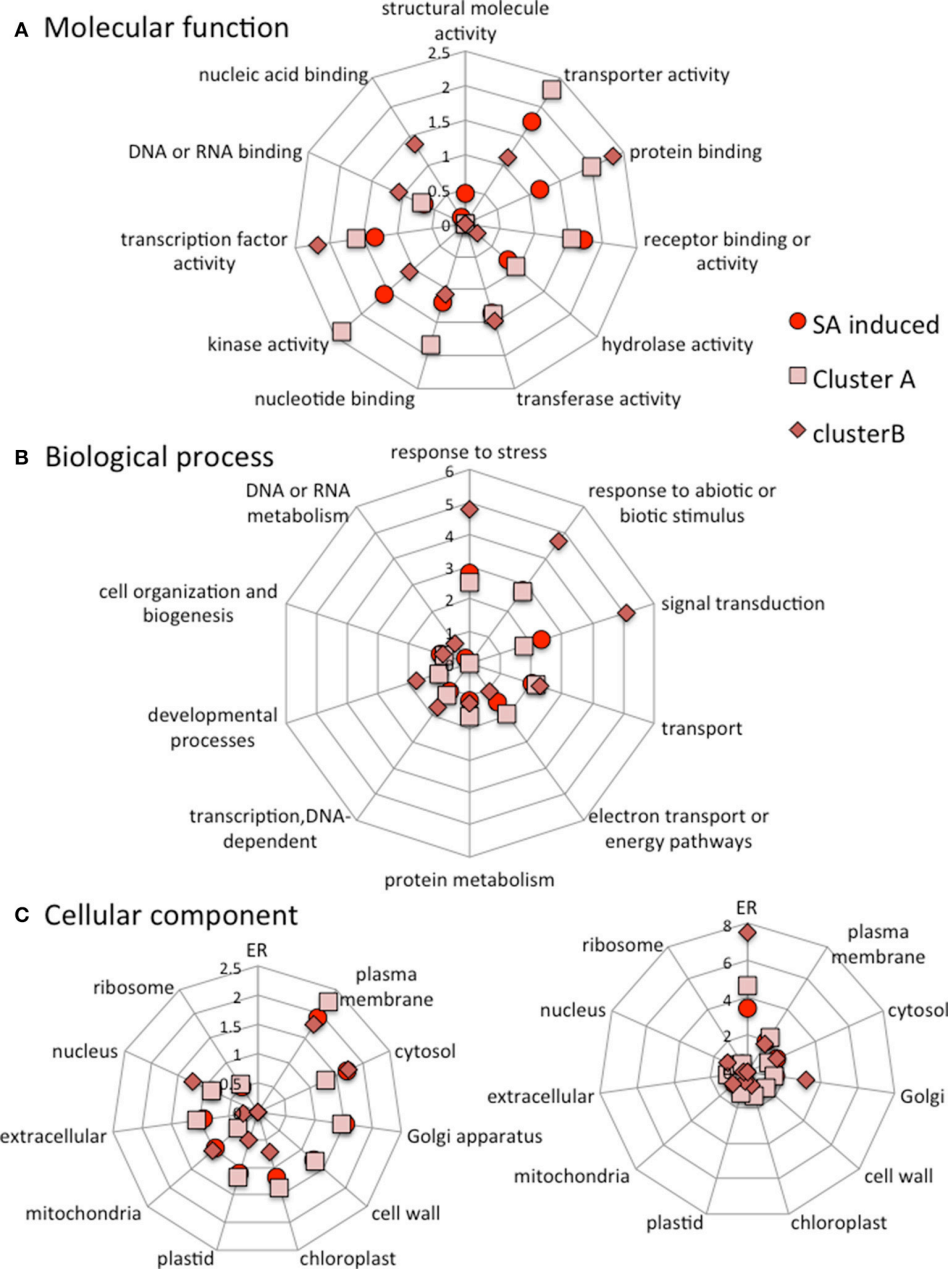
**Categorization of all SA-induced genes, cluster A genes and cluster B genes according to the molecular functions (A), Biological processes (B) and Cellular components (C) they are associated with**. The data are normalized to category frequencies in the Arabidopsis genome dataset. The mean and *SD* for 100 bootstraps of the input were calculated. For cellular component analysis, two scales are used for clarity.

The stringent clusters of genes are selected subsets of genes induced or repressed by SA. Some of the gene names from the four clusters are listed in Figure 7 with more precise details of their individual expression profiles. The genes of the different stringent clusters were classified according to gene ontology (GO) classes of “Biological processes,” “Molecular function,” and “Cellular component.” The results were normalized to the frequency of each class over the entire genome (Figure 8).

When compared to all SA-induced genes, cluster A is poor in genes involved in “response to abiotic or biotic stress” biological processes and has less “structural molecule activity” but more “nucleic acid binding” molecular functions. Cluster B is enriched in genes involved in “response to stress,” “response to abiotic and biotic stress” and “signal transduction” biological processes. Cluster B is also depleted in “nucleotide binding” and “transcription factor activity,” but is enriched in “protein binding” molecular functions. Cluster B is enriched in “endoplasmic reticulum” and “Golgi” cellular components but depleted in “cell wall,” “extracellular” and “ribosome” cellular components (Figure 8).

**Figure 9:**
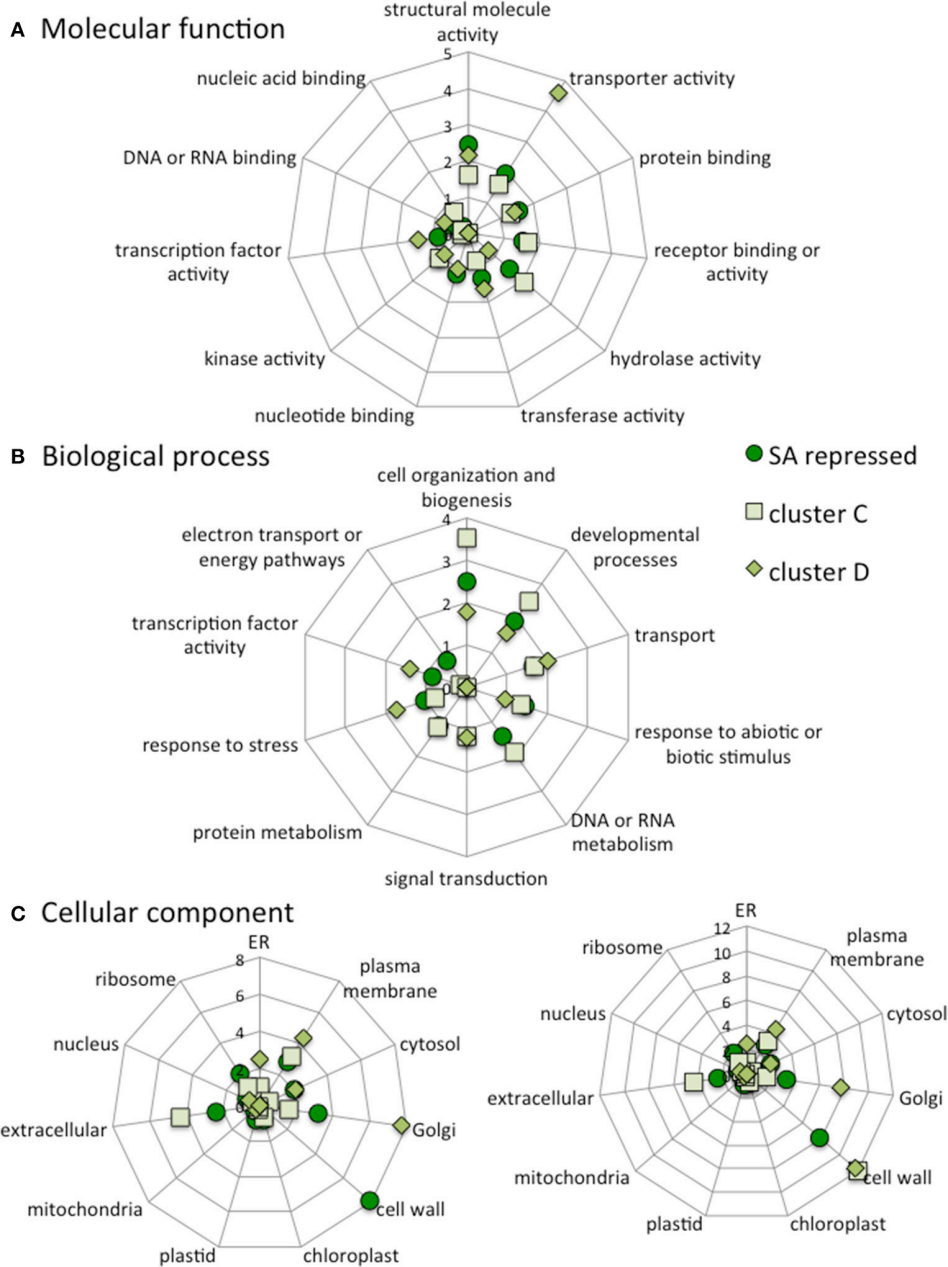
**Categorization of all SA-repressed genes, cluster C genes and cluster D genes according to the molecular functions (A), Biological processes (B) and Cellular components (C) they are associated with**. The data are normalized to category frequencies in the Arabidopsis genome dataset. The mean and *SD* for 100 bootstraps of the input were calculated. For cellular component analysis, two scales are used for clarity.

When compared to all SA-repressed genes in ACSC, cluster C is depleted in genes involved in “developmental” and “electron transport” biological processes and in “nucleotide binding” and “transcription factor activity” molecular functions. Cluster C is enriched in genes encoding “cell wall” and “extracellular” components but is depleted in “plastid” components. Cluster D is depleted in genes involved in “cell organization,” “responses to abiotic and biotic stresses,” “DNA or RNA metabolism,” “protein metabolism” and “electron transport” biological processes. Cluster D is also depleted in “receptor binding activity” and “hydrolase activity” molecular functions and in “plasma membrane” and “extracellular” cellular components (Figure 9).

### Specific promoter cis-elements are over-represented in the different clusters

**Figure 10:**
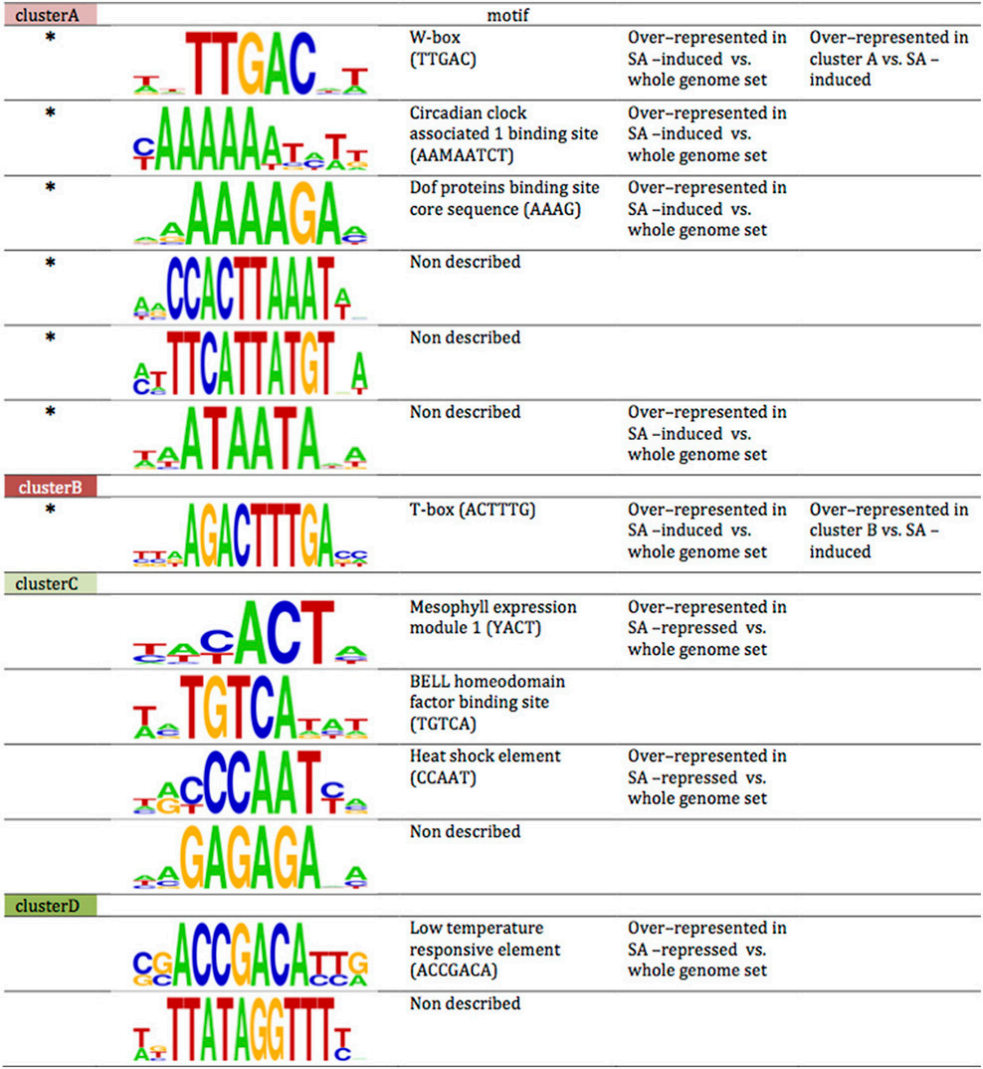
**Motifs over-represented in clusters A, B and C, D compared to whole genome promoter set**. Sequences of promoter regions were analyzed for 4- to 10-bp motifs over-represented in genes of the cluster vs. the whole genome set (*p*-value < 10^−5^; Chi-squared test). Among the motifs thus obtained a search for described *cis*-acting elements was performed. ^*^, cis-acting element is located on reverse strand; M, A or C; Y, C or T. Cis-acting element is over-represented compared to the bulk list of SA-induced genes. The sizes of the nucleotide symbols indicate their frequency in the corresponding sequence.

The transcriptome responses to SA and to PI-PLC inhibition are rapid. We compared the promoters of the genes in *stringent* clusters A, B, C, and D by scanning for motifs. Some motifs are over-represented in the genes of the clusters relative to the whole genome dataset (Figure 10). Some of the motifs identified in clusters A and B and in clusters C and D are also over-represented when the motif scan compared all SA-induced and SA-repressed genes against the whole genome data set. This is not surprising since these clusters together include most of the SA-sensitive genes of Arabidopsis. In cluster A promoters, the W box is over-represented compared to the whole set of SA induced genes. In cluster B promoters, the T-box motif is over-represented when compared to the whole set of SA-induced genes. Not all the sequence motifs found are known as regulatory elements.

### The genes of the different clusters differ in their SA specificity

**Figure 11:**
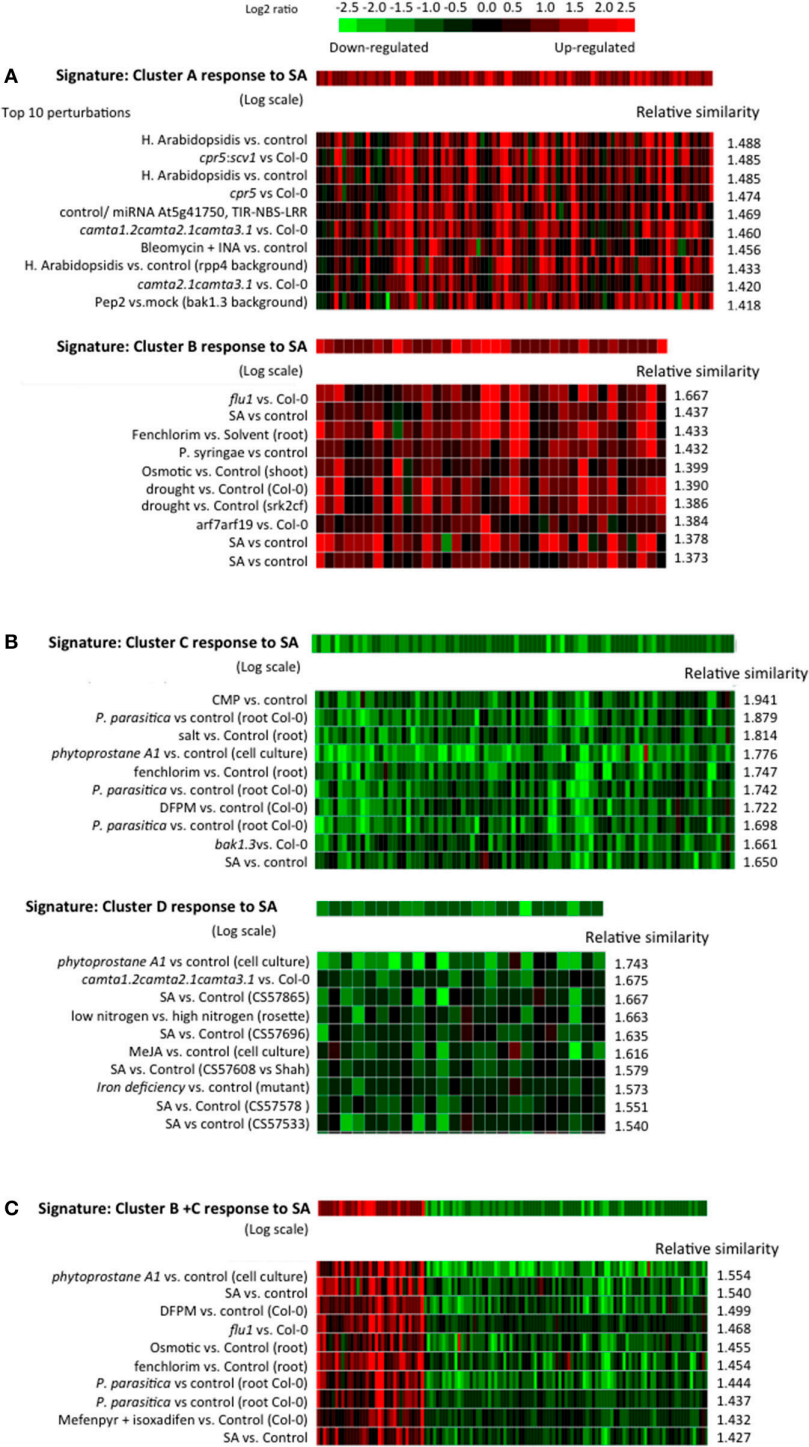
**Similarity between the SA responses of cluster A, B, C, and D and archived Arabidopsis transcriptomes in public databases**. **(A)** Top 10 similar experiments retrieved using the response to SA of cluster A or cluster B genes as a signature. **(B)** Top 10 similar experiments retrieved using the response to SA of cluster C or cluster D genes as a signature. **(C)** Top 10 similar experiments retrieved using, as a signature, the response to SA of a list of genes composed of cluster B and C. The similarity search was performed using the Genevestigator signature module. An Euclidean distance-derived similarity score was calculated between our signature and each experiment of a set. A relative similarity score was calculated where a relative similarity score of 1 stands for a similarity between the input signature and an experiment that is the same as the average over all experiments of the set.

More signature similarity searches were carried out with the genes of each of the different clusters. Which conditions lead to a similar shift in gene expression as the expression of each cluster in response to SA (Figure 11)?

For the signature SA response of cluster A, the top 10 most similar perturbations all relate to biotic stress and/or SA. For example, similar transcriptomes include Arabidopsis treated with: *Hyeloperonospora arabidopsidsis* (the oomycete causal agent of downy mildew of Arabidopsis); bleomycin, an iron chelator (iron chelation is known to cause an increase in SA, Dellagi et al., 2009); pep2, an Arabidopsis derived active peptide elicitor that facilitates immune signaling and pathogen defense responses (Ross et al., 2014); or SA. Other similar transcriptomes were found in comparisons of *cpr5* (Bowling et al., 1994, 1997), and a *camta* triple mutant (Kim et al., 2013), mutants with constitutively high endogenous SA, to wild type plants. The signature of the cluster B response to SA does not match the same gene expression profiles as cluster A except for experiments dealing with treatment with SA. The top 10 most similar physiological perturbations of Arabidopsis include: inoculation with *Pseudomonas syringae*, a biotic stress that induces SA; treatment with fenclorim (4,6-dichloro-2-phenylpyrimidine), a safener that increases tolerance of chloroacetanilide herbicides in rice by enhancing the expression of detoxifying glutathione S-transferases (Brazier-Hicks et al., 2008; Skipsey et al., 2011); osmotic stress; and drought. Genetic perturbations with similar gene expression effects are *flu1* or *arf7arf19* compared to wild type plants. *FLU1* encodes a coiled-coil, TPR domain-containing protein that is localized to the chloroplast membrane and is involved in chlorophyll biosynthesis. *flu1* plants accumulate protochlorophyllide, an intermediate in the chlorophyll biosynthesis pathway and release singlet oxygen in plastids upon a dark/light shift (Laloi et al., 2006). *arf7arf19* is a loss-of-function mutant of the auxin response factors ARF7 and ARF19 (Narise et al., 2010). Therefore, the genes of cluster B, while responding to SA, appear to be less characteristic of a typical SA response, and might correspond to a more general stress response like the oxidative stress response.

The response of cluster C genes to SA is similar to other SA or biotic stress experiments, but also to responses of plants treated with: salt; fenchlorim; phytoprostane A1, which is a cyclopentenone oxylipin (Stotz et al., 2013); and DFPM ([5-(3,4-dichlorophenyl)furan-2-yl]-piperidine-1-ylmethanethione), a small molecule that rapidly down-regulates ABA-dependent gene expression (Kim et al., 2011). Here again, the gene response signature seems not to be specific to SA. For the cluster D response to SA, most of the similarities are to gene expression profiles in plants treated with SA or having high endogenous amounts of SA.

It is intriguing to find that the response to SA of genes of cluster B (SA-induced) and cluster C (SA-repressed) retrieve some of the same experiments in the signature searches. We therefore built an artificial list of genes merging clusters B and C. The response to SA of these genes was used as a signature in a similarity search and again SA experiments were the most similar, confirming that using either induced or repressed genes alone does not distort the accuracy of signature searches.

### SA responses of the clusters differ in their dependency on PLD

We have previously shown that SA activates PLD activity in ACSC (Krinke et al., 2009). We wanted to know if any genes of the different clusters depend on PLD for their response to SA. PLD can use primary alcohols as a substrate, producing phosphatidyl-alcohol instead of PA. It is therefore possible to identify which genes are dependent on PLD for their expression by studying the effect of primary alcohol (e.g., *n*-butanol, *n*-ButOH) and comparing it to the effect of a non-substrate alcohol (*tertiary*-butanol, *tert*-ButOH) (Krinke et al., 2009). We thus showed that for some genes their response to SA could be inhibited by *n*-butanol, suggesting that the SA response is dependent on PLD activity.

**Figure 12:**
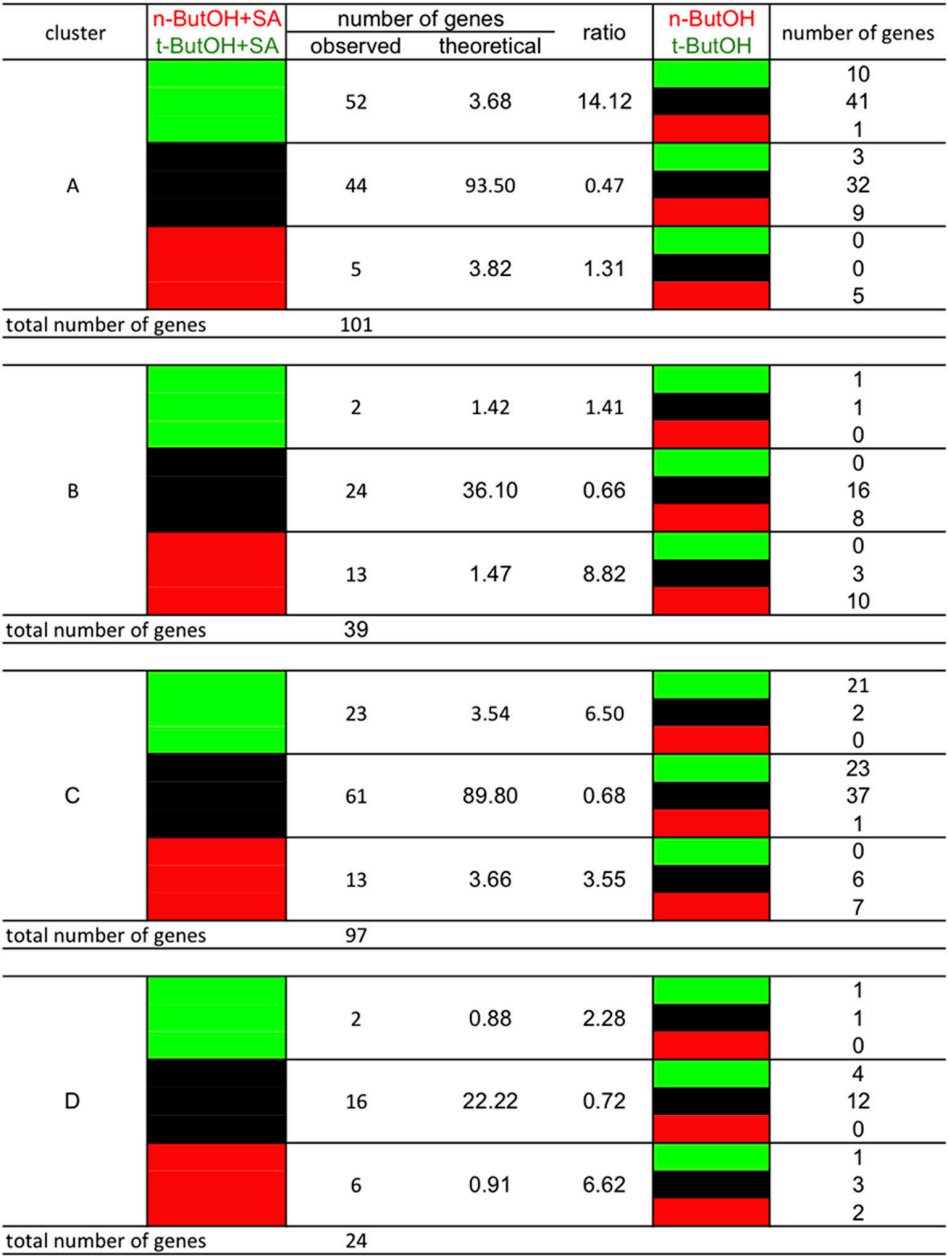
**Crosstalk in gene expression in response to SA and PI-PLC dependency as defined by the clusters and dependency on PLD activity**. The analysis was performed using stringent clusters as defined in the main text. Red blocks indicate relative higher transcript levels in the condition written in red at the top of the table (versus that written in green); green blocks indicate higher transcript levels in the conditions written green at the top of the table (versus that written in red) and black blocks indicate no significant difference in transcript levels between both conditions.

The genes of the *stringent* clusters A, B, C, and D were sorted according to the effect of *n*-ButOH compared to *tert*-ButOH in the presence or absence of SA (Figure 12). If we first consider the SA-induced genes, we see a marked difference between clusters A and B. In cluster A, the SA induction of many more genes is repressed by *n*-ButOH rather than enhanced. There is a 14-fold over-representation in *stringent* cluster A of genes whose induction by SA is repressed by *n*-ButOH compared to *tert*-ButOH. On the contrary, for *stringent* cluster B genes, there is an over-representation of genes for which *n*-ButOH enhances the induction by SA. The effect of *n*-ButOH is seen even on the basal expression of cluster B genes. For genes inhibited by SA, in both *stringent* clusters C and D, the differences in the effect of *n*-ButOH or *tert*-ButOH on SA was not as marked as for cluster A genes.

### Genes of the different clusters differ in their response to R59022, a DGK inhibitor

**Figure 13:**
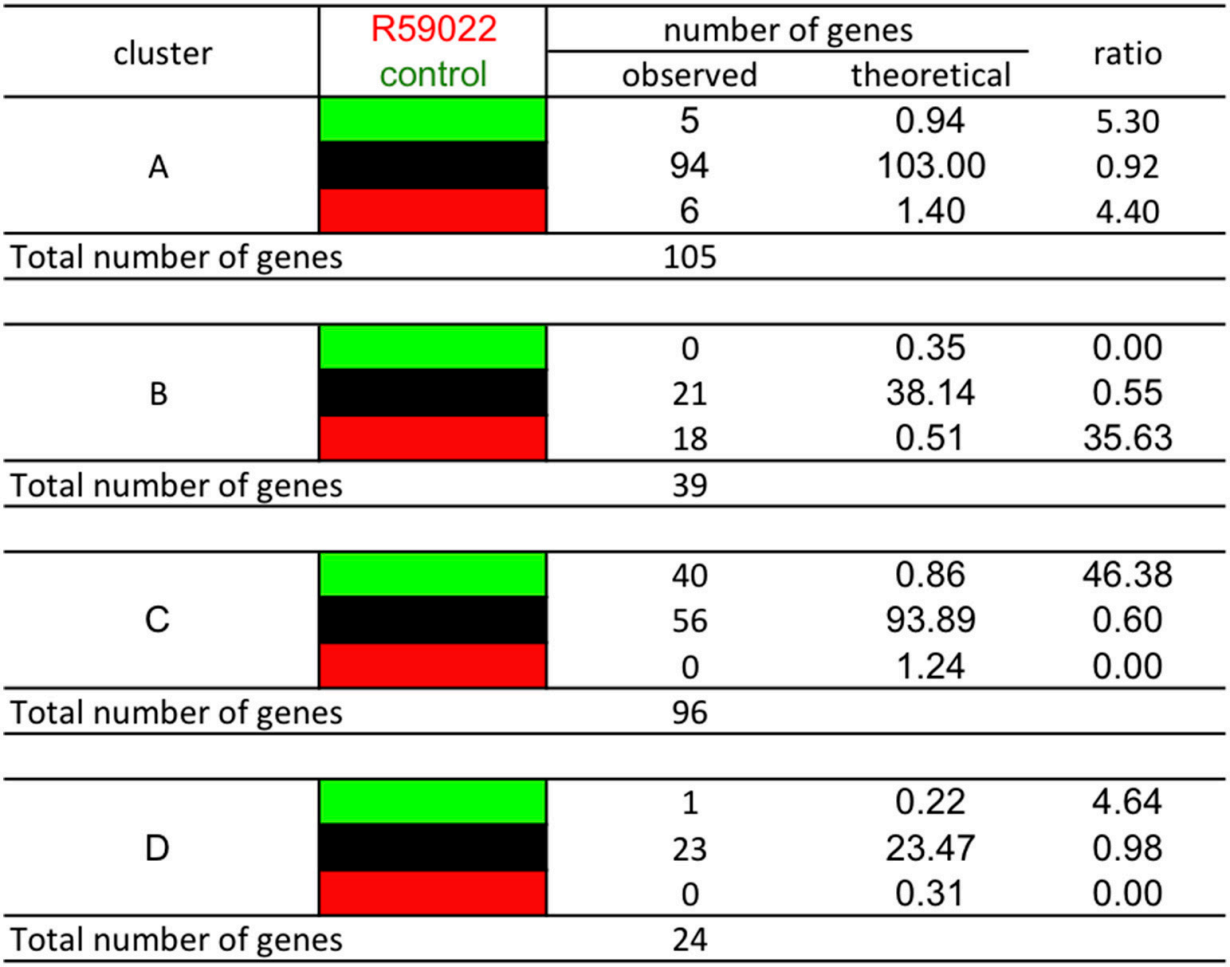
**Classification of the genes of the different clusters according to their response to DGK inhibitor R59022**. The analysis was performed using stringent clusters as defined in the main text. Red blocks indicate relative higher transcript levels in the condition written in red at the top of the table (versus that written in green); green blocks indicate higher transcript levels in the conditions written green at the top of the table (versus that written in red) and black blocks indicate no significant difference in transcript levels between both conditions.

The effect of basal PI-PLC can be partially attributed to a secondary effect on basal DGK, as seen by the action of DGK inhibitor R59022 (Djafi et al., 2013). Each gene of the different stringent clusters was sorted according to its expression in response to R59022 (Figure 13). Knowing that in the total group of genes 181 genes were repressed, 19705 genes were unaffected, and 261 genes were induced by R59022, we calculated the theoretical number of genes that should be present in each cluster if the gene expression was independent from the other cluster criteria.

Amongst cluster B genes, there is a 36-fold over-representation of genes that are induced by R59022. The basal level of these genes is negatively regulated by DGK. Conversely, amongst cluster C genes, there is a 31-fold over-representation of genes that are repressed by R59022. The basal level of those genes is positively regulated by DGK.

## Discussion

In a previous work we detected basal PI-PLC activity *in vivo* by assaying the DGK activity that is most likely coupled to this PI-PLC. PI-PLC was active in ACSC and this activity controlled the expression of some genes. To draw up the list of genes controlled by the basal PI-PLC activity, edelfosine, and U73122 were used separately to inhibit PI-PLC activity. The limitations of such a pharmacological approach were already discussed in depth (Djafi et al., 2013), but need to be taken into account here. Briefly, U73122, the most commonly used PI-PLC inhibitor, might have side effects. It is thought to be an alkylating agent and certain side effects might be attributable to general protein alkylation (Mogami et al., 1997; Horowitz et al., 2005). We previously demonstrated that edelfosine inhibits PI-PLC activation by cold shock in ACSC (Ruelland et al., 2002). Edelfosine does not have the structure of an alkylating agent and does not have the same side effects as U73122 (Powis et al., 1992; Horowitz et al., 2005; Wong et al., 2007; Kelm et al., 2010). It could have other side effects, since its lyso-alkyl-phosphatidylcholine structure makes it an inhibitor of CTP:phosphocholine cytidylyltransferase (Boggs et al., 1995). However, we have shown that these two unrelated molecules have similar inhibitory effects on the same enzyme in a pathway leading to the same changes in gene expression.

Similarity searches for gene expression signatures provide preliminary indications that edelfosine and U73122 trigger transcriptome changes similar to those induced by SA in whole seedlings, leaves and ACSC (Krinke et al., 2007). More genes are regulated, whether repressed or induced, in the same way by SA and PI-PLC inhibitors than would be expected if the effects were independent. Interestingly, Chou et al. (2004) identified a few genes for which inhibition of PI-PLC by U73122 mimics infection by *Pseudomonas syringae*. Their conclusion that inhibition of PI-PLC results in the induction of pathogenesis-related genes is validated by our results (Chou et al., 2004).

Using archived microarray data from our model system of ACSC treated with or without SA and applying stringent criteria, we defined four gene clusters. Clusters A and B are SA-induced and clusters C and D are SA-repressed. Clusters A and D define a pool of genes dependent on phosphoinositides for their SA response, while clusters B and C define a pool of genes dependent on PI-PLC products for their SA response. The clusters are not random subsets of the complete sets of SA-induced and SA-repressed genes. Based on their promoter sequences and GO classifications they represent distinct subsets, particularly clusters B and C. For instance, the GO “cell wall” term within the cellular component category is 12-fold more frequent in clusters C and D than in the whole genome, but only 8-fold more frequent in the SA-repressed genes. By contrast the “cell wall” term does not appear at all in cluster B gene descriptions. For the biological processes, “cell organization and biogenesis” and “developmental process” are enriched in cluster C, SA-repressed and cluster D genes (in that order), but not in cluster, A, cluster B or SA-induced genes. On the other hand, while SA-induced genes and cluster A genes are similarly enriched in genes involved in “response to stress,” “response to abiotic or biotic stimulus” and “signal transduction,” cluster B includes roughly twice as many genes from these categories. So clearly, the downstream cellular processes controlled by each gene cluster are distinct.

When the SA responses of genes in the different clusters were used in similarity searches, the genes of clusters A and D define different signatures than those of clusters B and C. The gene responses of clusters A and D are quite SA-specific. This is well characterized by the presence of *PR-1*, the most studied SA responsive gene, in cluster A (Krinke et al., 2007). Clusters B and C form a common pool of genes because their responses to SA are mimicked by edelfosine, by U73122, by wortmannin and to a certain extent by R59022. The responses of genes in clusters B and C are not only characteristic of SA treatment, as expected, but also of treatments with herbicide safeners, or to osmotic stress. When the genes of clusters B and C are merged into a single list, the same microarray experiments with similar gene responses are retrieved, showing that these two clusters define a specific pool of SA responsive genes. The specificities of the pools hold true for the crosstalk between PLD and DGK. Genes for which the effect of DGK inhibitor is similar to the effect of SA (and incidentally of W30 and inhibitors of PI-PLC) are over-represented in clusters B and C. For clusters A and D, no such distortion is seen in the DGK inhibitor effect.

Clusters A and B represent 23 and 8% of SA-induced genes, respectively, while clusters C and D represent 31 and 7% of SA-repressed genes. However, the sizes of these clusters are certainly underestimated. Due to the transcriptome analysis thresholds used, it is likely that some genes have not been classified as being induced or repressed by a particular treatment, even though they were induced or repressed. For clustering, the more criteria are considered, the more genes are mistakenly discounted. This is especially true for cluster B and C genes, which were scored as being responsive to at least four molecules (SA, W30, U73122, and edelfosine). It is possible to define looser clusters by considering that a gene belongs to a cluster if it is affected by only one of the two PI-PLC inhibitors. This returns 121, 113, 164, and 27 genes for *loose* clusters A, B, C, and D, respectively (see Table S5). In this case, clusters A and B represent 26 and 24% of SA-induced genes, respectively, while clusters C and D represent 52 and 9% of SA-repressed genes. It is perhaps important to note that cluster C represents most of the SA-repressed transcriptome. In Figure 5 we defined clusters B^E^ and B^W30^ as the genes that would belong to cluster B if we had considered that a basal inhibiting effect of either edelfosine or W30, respectively, was sufficient for a gene to be classified as cluster B (see Figure 5). Similarly, we defined cluster C^E^ and C^W30^ as genes that have the same characteristics as cluster C genes, but are repressed by only edelfosine or W30, respectively. Genes in clusters B^E^ and B^W30^ and in C^E^ and C^W30^ might be sorted as B and C respectively if slightly different threshold criteria were used for microarray analysis. If the genes of clusters B^E^, B^W30^, C^E^, and C^W30^ were considered in this way, the percentage of B and C cluster genes among SA-induced or SA-repressed genes would be even higher. The effect of SA inhibition of PI-PLC on gene expression appears to be a major event in the SA response.

What is the significance of the pool of genes in clusters B and C? What is the SA-triggered signaling event controlling expression of this pool? From a lipid signaling point of view it is tempting to consider that these genes are regulated in resting cells by PI-PLC pathway products. However, correlation alone is not proof. For clusters B and C we have only described correlations between different datasets showing that (i) challenging cells with SA induces *in vivo* PI-PLC inhibition, and (ii) inhibiting PI-PLC by different drugs alters the expression of a set of genes that is also affected when SA was added. We cannot rule out the possibility that the common effect of SA, wortmannin, and PI-PLC inhibitors on the same gene is due to another mechanism. To date, the inhibition of the PI-PLC pathway proposed in our working model seems the most obvious mechanism (Figure 3). Molecular events need to be pinpointed more precisely to make a firmer conclusion. Experimentally, one possibility would be to inhibit the PI-PLC inhibition by over-expressing PI-PLC. However, even over-expressed PI-PLC activity might be subject to the inhibition. We can be more confident that PI-PLC activity regulates expression of genes of clusters A and D, as these genes were specifically selected because their response to SA is inhibited when W30 blocks the accumulation of phosphoinositide.

Where does PI-PLC fit in SA signaling *in planta*? In radiolabeled ACSC, we showed that SA induces an increase in phosphoinositides, the substrates of PI-PLC, and a decrease in PA, a derivative of PI-PLC product. This is indeed what is expected if SA inhibits PI-PLC *in vivo*. We showed previously that the phosphoinositide increase required active type-III PI4K, which can be inhibited with wortmannin (Krinke et al., 2007). The type-III PI4Ks are the very ones that provide PI-PLC with substrates in response to cold (Delage et al., 2012). The basal PI-PLC activity is also fed with substrates formed by wortmannin-sensitive PI4K (Djafi et al., 2013). Therefore, the fact that SA activates a PI4K is compatible with it also inhibiting PI-PLC. It is likely that to monitor the phosphoinositide increase, it will be necessary to stimulate type-III PI4K activity and inhibit PI-PLC. Expressing human phosphatidylinositol phosphate kinase in tobacco resulted in a 40-fold increase in basal InsP_3_, while PI4P remained constant and PI(4,5)P_2_ increased 7-fold (Im et al., 2007). This suggests that if basal PI-PLC is not inhibited, an increase in the formation of its substrates would directly lead to more products, which is not what we observe. Finally it is important to note that independent data suggests that SA can inhibit PI-PLC. In *Capsicum chinense* J. cells, treatment with SA led to inhibition of PI-PLC but increased lipid kinase activities measured *in vitro* (Altuzar-Molina et al., 2011), which is consistent with our observed data. How could SA inhibit PI-PLC? The effect of SA on phosphoinositide increase can be detected as early as 15 min after its addition to cell culture medium, so the action of SA is probably not due to transcriptional control. Besides, using the microarray data we can see that after 4 h of SA incubation, PI-PLC genes are not repressed by the hormone (PLC10 is slightly induced) and type III-PI4K and DGK genes are not affected by it (data not shown). Structurally, it is difficult to conceive how SA could act on PI-PLC directly. Plant PI-PLCs are structurally similar to the simplest mammal PI-PLC, but the way they are regulated is not documented (Pokotylo et al., 2014). It is conceivable that they are subject to post-translational modifications as some phosphorylation sites have been detected (Janda et al., 2013).

## Conclusion

We show that SA induces an increase in phosphoinositides with no increase in PA, the phosphorylated derivative of DAG, a PI-PLC product. This is likely to reflect an *in vivo* inhibition of PI-PLC by SA. We identified two pools of SA-responsive genes, one regulated by an increase in phosphoinositides and the other possibly by a decrease in PI-PLC products. Inhibition of PI-PLC by SA is likely to be a major step in the gene response to SA, especially in regulation of SA-inhibited genes, at least in ACSC. At a time when a great deal of omics data are being generated, this study illustrates that fine data mining using pertinent tools can reveal important processes.

## Materials and methods

### Cell culture, labeling, and lipid analysis

*Arabidopsis thaliana* Col-0 suspension cells were cultivated as described by Krinke et al. (2009). Experiments were performed on 5-day-old cultures. Suspension cells were labeled with ^33^P_i_ according to the procedure previously described by Krinke et al. (2007). Total lipids were extracted and separated by thin layer chromatography (TLC). Structural phospholipids and PA were separated in the acid solvent system composed of chloroform-acetone-acetic acid-methanol-water (10:4:2:2:1, v/v/v/v/v) (Lepage, 1967). Phosphoinositides were separated in the alkaline solvent system composed of chloroform-methanol-5% (w/v) ammonia solution (9:7:2, v/v/v), where the TLC plates were soaked in potassium oxalate solution before heat activation (Munnik et al., 1994). Radiolabeled spots were quantified by autoradiography using a Storm Phosphorimager (Amersham Biosciences). Separated phospholipids were identified by co-migration with authentic non-labeled standards visualized by primuline staining (under UV light) or by phosphate staining.

### Transcriptomic data

No new transcriptomic data were generated for this study. The microarray data used for this article are deposited in Gene Expression Omnibus (http://www.ncbi.nlm.nih.gov/geo/; accession no. GSE7495, GSE9695, GSE19850 and GSE 35872) and CATdb (http://urgv.evry.inra.fr/CATdb/; Projects: RS05-04, AU07-01, RS09-04, and AU10-12).

### RNA extraction and transcript level

The extraction of RNA and the detection and quantification of transcripts were performed as in Djafi et al. (2013).

### Motif analysis

Promoters (up to -1000 bp upstream of the transcription start site, without overlaps with other genes, and excluding 5′UTRs) of genes were extracted from the database of The Arabidopsis Information Resource (TAIR; Rhee et al., 2003). Sequences were used to search for over-represented motifs ranging from 4 to 10 bp using SIFT software (Hudson and Quail, 2003). The list of genes used as reference was either the list of promoters from the whole genome (33,062 promoters) or from all SA-induced genes or SA-repressed genes, as indicated. The motifs we defined were then compared with the ones in the PLACE database (Higo et al., 1999) to search for related *cis*-elements and similar motifs.

### Conflict of interest statement

The authors declare that the research was conducted in the absence of any commercial or financial relationships that could be construed as a potential conflict of interest.

## References

[B1] Achard P. Genschik P. (2008). Releasing the brakes of plant growth: how GAs shutdown DELLA proteins. J. Exp. Bot. 60, 1085–1092. 10.1093/jxb/ern301 19043067

[B2] Altuzar-Molina A. R. Armando Munoz-Sanchez J. Vazquez-Flota F. Monforte-Gonzalez M. Racagni-Di Palma G. Teresa Hernandez-Sotomayor S. M. (2011). Phospholipidic signaling and vanillin production in response to salicylic acid and methyl jasmonate in Capsicum chinense. Plant Physiol. Biochem. 49, 151–158. 10.1016/j.plaphy.2010.11.005 21147536

[B3] Boggs K. P. Rock C. O. Jackowski S. (1995). Lysophosphatidylcholine and 1-O-octadecyl-2-O-methyl-rac-glycero-3-phosphocholine inhibit the CDP-choline pathway of phosphatidylcholine synthesis at the CTP:phosphocholine cytidylyltransferase step. J. Biol. Chem. 270, 7757–7764. 7706325 10.1074/jbc.270.13.7757

[B4] Bowling S. A. Clarke J. D. Liu Y. Klessig D. F. Dong X. (1997). The cpr5 mutant of Arabidopsis expresses both NPR1-dependent and NPR1-independent resistance. Plant Cell 9, 1573–1584. 9338960 10.1105/tpc.9.9.1573 PMC157034

[B3a] Bowling S. A. Guo A. Cao H. Gordon A. S. Klessig D. F. Dong X. I. (1994). A mutation in arabidopsis that leads to constitutive expression of systemic acquired-resistance. Plant Cell 6, 1845–1857. 7866028 10.1105/tpc.6.12.1845 PMC160566

[B5] Brazier-Hicks M. Evans K. M. Cunningham O. D. Hodgson D. R. W. Steel P. G. Edwards R. (2008). Catabolism of glutathione conjugates in *Arabidopsis thaliana*. Role in metabolic reactivation of the herbicide safener fenclorim. J. Biol. Chem. 283, 21102–21112. 10.1074/jbc.M801998200 18522943 PMC3258958

[B6] Chou W.-M. Shigaki T. Dammann C. Liu Y.-Q. Bhattacharyya M. K. (2004). Inhibition of phosphoinositide-specific phospholipase C results in the induction of pathogenesis-related genes in soybean. Plant Biol. (Stuttg.) 6, 664–672. 10.1055/s-2004-830351 15570470

[B7] Delage E. Ruelland E. Guillas I. Zachowski A. Puyaubert J. (2012). Arabidopsis Type-III Phosphatidylinositol 4-Kinases β 1 and β 2 are upstream of the phospholipase C pathway triggered by cold exposure. Plant Cell Physiol. 53, 565–576. 10.1093/pcp/pcs011 22318862

[B8] Dellagi A. Segond D. Rigault M. Fagard M. Simon C. Saindrenan P.. (2009). Microbial siderophores exert a subtle role in Arabidopsis during infection by manipulating the immune response and the iron status. Plant Physiol. 150, 1687–1696. 10.1104/pp.109.138636 19448037 PMC2719128

[B9] Djafi N. Vergnolle C. Cantrel C. Wietrzyñski W. Delage E. Cochet F.. (2013). The Arabidopsis DREB2 genetic pathway is constitutively repressed by basal phosphoinositide-dependent phospholipase C coupled to diacylglycerol kinase. Front. Plant Sci. 4:307. 10.3389/fpls.2013.00307 23964284 PMC3737466

[B9a] Durrant W. E. Dong X. (2004). Systemic acquired resistance. Annu. Rev. Phytopathol. 42, 185–209. 10.1146/annurev.phyto.42.040803.140421 15283665

[B10] Gutiérrez J. González-Pérez S. García-García F. Daly C. T. Lorenzo O. Revuelta J. L.. (2014). Programmed cell death activated by Rose Bengal in *Arabidopsis thaliana* cell suspension cultures requires functional chloroplasts. J. Exp. Bot. 65, 3081–3095. 10.1093/jxb/eru151 24723397 PMC4071827

[B11] Haapalainen M. Dauphin A. Li C.-M. Bailly G. Tran D. Briand J.. (2012). HrpZ harpins from different Pseudomonas syringae pathovars differ in molecular interactions and in induction of anion channel responses in *Arabidopsis thaliana* suspension cells. Plant Physiol. Biochem. 51, 168–174. 10.1016/j.plaphy.2011.10.022 22153254

[B12] Hallouin M. Ghelis T. Brault M. Bardat F. Cornel D. Miginiac E.. (2002). Plasmalemma abscisic acid perception leads to RAB18 expression via phospholipase D activation in Arabidopsis suspension cells. Plant Physiol. 130, 265–272. 10.1104/pp.004168 12226506 PMC166559

[B13] Higo K. Ugawa Y. Iwamoto M. Korenaga T. (1999). Plant cis-acting regulatory DNA elements (PLACE) database: 1999. Nucleic Acids Res. 27, 297–300. 9847208 10.1093/nar/27.1.297 PMC148163

[B14] Horowitz L. F. Hirdes W. Suh B.-C. Hilgemann D. W. Mackie K. Hille B. (2005). Phospholipase C in living cells activation, inhibition, Ca2+ requirement, and regulation of M current. J. Gen. Physiol. 126, 243–262. 10.1085/jgp.200509309 16129772 PMC2266577

[B14a] Horvath E. Szalai G. Janda T. (2007). Induction of abiotic stress tolerance by salicylic acid signaling. J. Plant Growth Regul. 26, 290–300 10.1007/s00344-007-9017-4

[B15] Hruz T. Laule O. Szabo G. Wessendorp F. Bleuler S. Oertle L.. (2008). Genevestigator v3: a reference expression database for the meta-analysis of transcriptomes. Adv. Bioinformatics 2008:420747. 10.1155/2008/420747 19956698 PMC2777001

[B16] Hudson M. E. Quail P. H. (2003). Identification of promoter motifs involved in the network of phytochrome A-Regulated gene expression by combined analysis of genomic sequence and microarray data. Plant Physiol. 133, 1605–1616. 10.1104/pp.103.030437 14681527 PMC300717

[B17] Im Y. J. Perera I. Y. Brglez I. Davis A. J. Stevenson-Paulik J. Phillippy B. Q.. (2007). Increasing plasma membrane phosphatidylinositol(4,5)bisphosphate biosynthesis increases phosphoinositide metabolism in *Nicotiana tabacum*. Plant Cell 19, 1603–1616. 10.1105/tpc.107.051367 17496116 PMC1913725

[B18] Janda M. Planchais S. Djafi N. Martinec J. Burketova L. Valentova O.. (2013). Phosphoglycerolipids are master players in plant hormone signal transduction. Plant Cell Rep. 32, 839–851. 10.1007/s00299-013-1399-0 23471417

[B19] Janda M. Ruelland E. (in press). Magical mystery tour: salicylic acid signalling. Environ. Exp. Bot. 10.1016/j.envexpbot.2014.07.003

[B20a] Kalachova T. A. Iakovenko O. M. Kretinin S. V. Kravets V. S. (2012). Effects of salicylic and jasmonic acid on phospholipase D activity and the level of active oxygen species in soybean seedlings. Biol. Membrany 29, 169–176 10.1134/S1990747812030099

[B20] Kelm M. K. Weinberg R. J. Criswell H. E. Breese G. R. (2010). The PLC/IP3R/PKC pathway is required for ethanol-enhanced GABA release. Neuropharmacology 58, 1179–1186. 10.1016/j.neuropharm.2010.02.018 20206640 PMC2849882

[B21] Kim T. H. Hauser F. Ha T. Xue S. Böhmer M. Nishimura N.. (2011). Chemical genetics reveals negative regulation of abscisic acid signaling by a plant immune response pathway. Curr. Biol. 21, 990–997. 10.1016/j.cub.2011.04.045 21620700 PMC3109272

[B22] Kim Y. Park S. Gilmour S. J. Thomashow M. F. (2013). Roles of CAMTA transcription factors and salicylic acid in configuring the low-temperature transcriptome and freezing tolerance of Arabidopsis. Plant. J. 75, 364–376. 10.1111/tpj.12205 23581962

[B23] Krinke O. Flemr M. Vergnolle C. Collin S. Renou J.-P. Taconnat L.. (2009). Phospholipase D activation is an early component of the salicylic acid signaling pathway in Arabidopsis cell suspensions. Plant Physiol. 150, 424–436. 10.1104/pp.108.133595 19304931 PMC2675726

[B24] Krinke O. Ruelland E. Valentová O. Vergnolle C. Renou J.-P. Taconnat L.. (2007). Phosphatidylinositol 4-kinase activation is an early response to salicylic acid in Arabidopsis suspension cells. Plant Physiol. 144, 1347–1359. 10.1104/pp.107.100842 17496105 PMC1914138

[B25] Kunz S. Pesquet E. Kleczkowski L. A. (2014). Functional dissection of sugar signals affecting gene expression in *Arabidopsis thaliana*. PLoS ONE 9:e100312. 10.1371/journal.pone.0100312 24950222 PMC4065033

[B26] Laloi M. Perret A.-M. Chatre L. Melser S. Cantrel C. Vaultier M.-N.. (2006). Insights into the role of specific lipids in the formation and delivery of lipid microdomains to the plasma membrane of plant cells. Plant Physiol. 143, 461–472. 10.1104/pp.106.091496 17114270 PMC1761958

[B27] Ledoux Q. Van Cutsem P. Markó I. E. Veys P. (2014). Specific localization and measurement of hydrogen peroxide in *Arabidopsis thaliana* cell suspensions and protoplasts elicited by COS-OGA. Plant Signal. Behav. 9:e28824. 10.4161/psb.28824 24736566 PMC4091596

[B28] Lepage M. (1967). Identification and composition of turnip root lipids. Lipids 2, 244–250. 17805774 10.1007/BF02532563

[B29a] Malamy J. Carr J. P. Klessig D. F. Raskin I. (1990). Salicylic-acid – a likely endogenous signal in the resistance response of tobacco to viral-infection. Science 250, 1002–1004. 10.1126/science.250.4983.1002 17746925

[B29b] Metraux J. P. Signer H. Ryals J. Ward E. Wyssbenz M. Gaudin J.. (1990). Increase in salicylic-acid at the onset of systemic acquired-resistance in cucumber. Science 250, 1004–1006. 10.1126/science.250.4983.1004 17746926

[B29] Mogami H. Lloyd Mills C. Gallacher D. V. (1997). Phospholipase C inhibitor, U73122, releases intracellular Ca^2+^, potentiates Ins(1,4,5)P3-mediated Ca^2+^ release and directly activates ion channels in mouse pancreatic acinar cells. Biochem. J. 324, 645–651. 9182729 10.1042/bj3240645 PMC1218477

[B30] Munnik T. Irvine R. Musgrave A. (1994). Rapid turnover of phosphatidylinositol 3-phosphate in the green-alga chlamydomonas-eugametos - signs of a phosphatidylinositide 3-kinase signaling pathway in lower plants. Biochem. J. 298, 269–273. 8135730 10.1042/bj2980269 PMC1137935

[B31] Narise T. Kobayashi K. Baba S. Shimojima M. Masuda S. Fukaki H.. (2010). Involvement of auxin signaling mediated by IAA14 and ARF7/19 in membrane lipid remodeling during phosphate starvation. Plant Mol. Biol. 72, 533–544. 10.1007/s11103-009-9589-4 20043234

[B32] Navarro L. Bari R. Achard P. Lisón P. Nemri A. Harberd N. P.. (2008). DELLAs control plant immune responses by modulating the balance of jasmonic acid and salicylic acid signaling. Curr. Biol. 18, 650–655. 10.1016/j.cub.2008.03.060 18450451

[B33] Pokotylo I. Kolesnikov Y. Kravets V. Zachowski A. Ruelland E. (2014). Plant phosphoinositide-dependent phospholipases C: variations around a canonical theme. Biochimie 96, 144–157. 10.1016/j.biochi.2013.07.004 23856562

[B34] Powis G. Seewald M. J. Gratas C. Melder D. Riebow J. Modest E. J. (1992). Selective inhibition of phosphatidylinositol phospholipase C by cytotoxic ether lipid analogues. Cancer Res. 52, 2835–2840. 1316230

[B34a] Profotova B. Burketova L. Novotna Z. Martinec J. Valentova O. (2006). Involvement of phospholipases C and D in early response to SAR and ISR inducers in Brassica napus plants. Plant Physiol. Biochem. 44, 143–151. 10.1016/j.plaphy.2006.02.003 16644231

[B35] Rainteau D. Humbert L. Delage E. Vergnolle C. Cantrel C. Maubert M.-A.. (2012). Acyl chains of Phospholipase D transphosphatidylation products in Arabidopsis cells: a study using multiple reaction monitoring mass spectrometry. PLoS ONE 7:e41985. 10.1371/journal.pone.0041985 22848682 PMC3405027

[B36] Rhee S.-Y. Beavis W. Berardini T. Z. Chen G. Dixon D. Doyle A.. (2003). The Arabidopsis Information Resource (TAIR): a model organism database providing a centralized, curated gateway to Arabidopsis biology, research materials and community. Nucleic Acids Res. 31, 224–228. 10.1093/nar/gkg076 12519987 PMC165523

[B37] Ross A. Yamada K. Hiruma K. Yamashita-Yamada M. Lu X. Takano Y.. (2014). The Arabidopsis PEPR pathway couples local and systemic plant immunity. EMBO J. 33, 62–75. 10.1002/embj.201284303 24357608 PMC3990683

[B38] Ruelland E. Cantrel C. Gawer M. Kader J.-C. Zachowski A. (2002). Activation of phospholipases C and D is an early response to a cold exposure in Arabidopsis suspension cells. Plant Physiol. 130, 999–1007. 10.1104/pp.006080 12376663 PMC166625

[B38a] Ruelland E. Kravets V. Derevyanchuk M. Martinec J. Zachowski A. I. Pokotylo I. (in press). Role of phospholipid signalling in plant environmental responses. Envir. Exp. Bot. 10.1016/j.envexpbot.2014.08.00917805774

[B39] Schweighofer A. Shubchynskyy V. Kazanaviciute V. Djamei A. Meskiene I. (2014). Bimolecular fluorescent complementation (BiFC) by MAP kinases and MAPK phosphatases. Methods Mol. Biol. 1171, 147–158. 10.1007/978-1-4939-0922-3_12 24908126

[B40] Seifertová D. Skùpa P. Rychtáø J. Laòková M. Pařezová M. Dobrev P. I.. (2014). Characterization of transmembrane auxin transport in Arabidopsis suspension-cultured cells. J. Plant Physiol. 171, 429–437. 10.1016/j.jplph.2013.09.026 24594395

[B41] Skipsey M. Knight K. M. Brazier-Hicks M. Dixon D. P. Steel P. G. Edwards R. (2011). Xenobiotic responsiveness of *Arabidopsis thaliana* to a chemical series derived from a herbicide safener. J. Biol. Chem. 286, 32268–32276. 10.1074/jbc.M111.252726 21778235 PMC3173150

[B42] Stotz H. U. Mueller S. Zoeller M. Mueller M. J. Berger S. (2013). TGA transcription factors and jasmonate-independent COI1 signalling regulate specific plant responses to reactive oxylipins. J. Exp. Bot. 64, 963–975. 10.1093/jxb/ers389 23349138 PMC3580818

[B43] Tjellström H. Yang Z. Allen D. K. Ohlrogge J. B. (2012). Rapid kinetic labeling of Arabidopsis cell suspension cultures: implications for models of lipid export from plastids. Plant Physiol. 158, 601–611. 10.1104/pp.111.186122 22128138 PMC3271753

[B44] Vaultier M.-N. Cantrel C. Vergnolle C. Justin A.-M. Demandre C. Benhassaine-Kesri G.. (2006). Desaturase mutants reveal that membrane rigidification acts as a cold perception mechanism upstream of the diacylglycerol kinase pathway in Arabidopsis cells. FEBS Lett. 580, 4218–4223. 10.1016/j.febslet.2006.06.083 16839551

[B44a] Vicente M. R. S. Plasencia J. (2011). Salicylic acid beyond defence: its role in plant growth and development. J. Exp. Bot. 62, 3321–3338. 10.1093/jxb/err031 21357767

[B45] Wong R. Fabian L. Forer A. Brill J. A. (2007). Phospholipase C and myosin light chain kinase inhibition define a common step in actin regulation during cytokinesis. BMC Cell Biol. 8:15. 10.1186/1471-2121-8-15 17509155 PMC1888687

