# Corrigendum: Salicylic acid modulates levels of phosphoinositide dependent-phospholipase C substrates and products to remodel the Arabidopsis suspension cell transcriptome

**DOI:** 10.3389/fpls.2016.00036

**Published:** 2016-01-28

**Authors:** Eric Ruelland, Igor Pokotylo, Nabila Djafi, Catherine Cantrel, Anne Repellin, Alain Zachowski

**Affiliations:** ^1^Université Paris-Est Créteil, Institut d'Ecologie et des Sciences de l'Environnement de ParisCréteil, France; ^2^Centre National de la Recherche Scientifique, Unité Mixte de Recherche 7618, Institut d'Ecologie et des Sciences de l'Environnement de ParisCréteil, France; ^3^Molecular Mechanisms of Plant Cell Regulation, Institute of Bioorganic Chemistry and Petrochemistry, National Academy of SciencesKyiv, Ukraine

**Keywords:** Arabidopsis, diacylglycerol kinase, trancriptomic, lipid Signaling, hormone transduction, salicylic acid, phospholipase C

In Figure 4 of the original article, an error was noticed. For the path leading to cluster C genes, wrong symbol “>” was used instead of “<.” One should read “Edel. < control; 137 genes.” Readers are referred to the Figure of this corrigendum, that replace the previous Figure 4.

**Figure 4:**
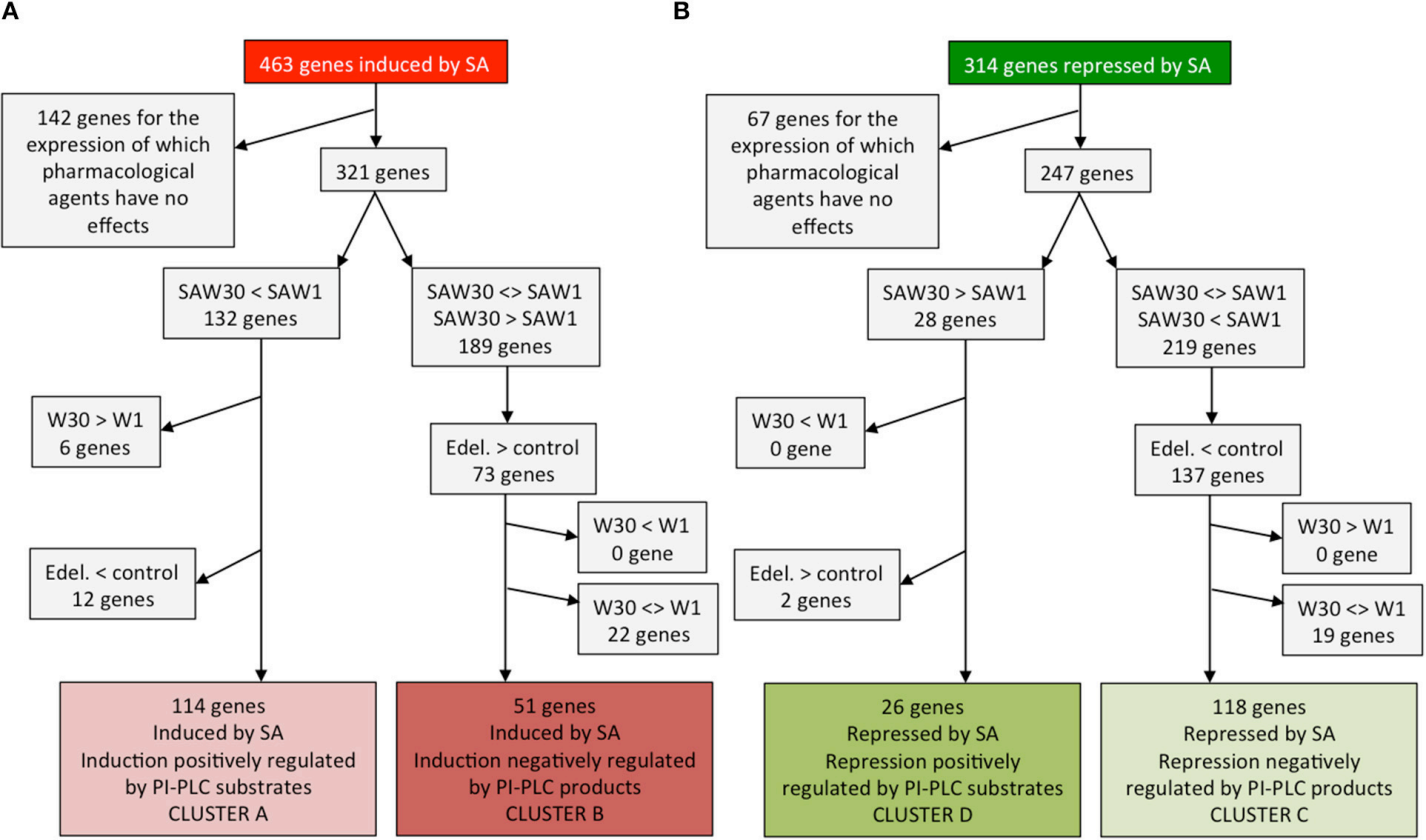
**Identification of genes whose expression characteristics are consistent with the clusters defined in Figure 3**. Cluster A and B genes are SA-induced genes **(A)** while clusters C and D genes are SA-repressed genes **(B)**.

## Conflict of interest statement

The authors declare that the research was conducted in the absence of any commercial or financial relationships that could be construed as a potential conflict of interest.

