# Supplementary material for: Salicylic acid modulates levels of phosphoinositide dependent-phospholipase C substrates and products to remodel the Arabidopsis suspension cell transcriptome
Source: Front Plant Sci. 2014 Nov 11;5:608. doi: 10.3389/fpls.2014.00608 (PMC4227474; doi:10.3389/fpls.2014.00608)
Supplement: Supplementary file 2 [file Table2.DOCX]

**Supplemental Table S2. Contingency table of the expression of genes to U73122 or SA treatments.** For each response mode, the number of genes previously reported to be upregulated (>), downregulated (<) or unaffected (=) by a treatment were included. Assuming that U73122 act independently of SA, the theotretical number of genes predicted to occur in each category (given in brackets) was calculated. The ratio of the “observed number of genes” to the “theoretical number of genes” is given in bold.

| Observed  (Theoretical)  **ratio** | SA> control | SA=control | SA< control | total |
| --- | --- | --- | --- | --- |
| U73122> U73343 | 107  (16.01)  **6.68** | 597  (678.13)  **0.88** | 1  (10.86)  **0.09** | 705 |
| U73122=U73343 | 360  (443.08)  **0.81** | 18934  (18760.53)  **1.01** | 210  (300.39)  **0.7** | 19504 |
| U73122< U73343 | 5  (12.90)  **0.39** | 454  (546.35)  **0.83** | 109  (8.75)  **12.46** | 568 |
| total | 472 | 19985 | 320 | 20777 |
